# TBD- or PS-TBD-Catalyzed One-Pot Synthesis of Cyanohydrin Carbonates and Cyanohydrin Acetates from Carbonyl Compounds

**DOI:** 10.3390/molecules21081030

**Published:** 2016-08-10

**Authors:** Satoru Matsukawa, Junya Kimura, Miki Yoshioka

**Affiliations:** Department of Science Education, Faculty of Education, Ibaraki University, Ibaraki 310-8512, Japan; varsil0403@gmail.com (J.K.); 13p1419r@vc.ibaraki.ac.jp (M.Y.)

**Keywords:** organocatalyst, basecatalyst, guanidine, cyanation, cyanoester

## Abstract

Cyanation reactions of carbonyl compounds with methyl cyanoformate or acetyl cyanide catalyzed by 5 mol % of 1,5,7-triazabicyclo[4,4,0]dec-5-ene (TBD) were examined. Using methyl cyanoformate, the corresponding cyanohydrin carbonates were readily obtained in high yield for aromatic and aliphatic aldehydes and ketones. Similar results were obtained when acetyl cyanide was used as the cyanide source. The polymer-supported catalyst, PS-TBD, also acted as a good catalyst for this reaction. PS-TBD was easily recovered and reused with minimal activity loss.

## 1. Introduction

The addition of cyanide to carbonyl compounds is among the most fundamental carbon–carbon bond formation reactions in modern organic chemistry [1,2,3,4,5,6]. The resultant cyanohydrins are easily converted into important synthetic intermediates including α-hydroxyl-carbonyl compounds and β-amino alcohols. Multiple cyanation methods have been examined, and trimethylsilyl cyanide has most commonly been used as the source of the cyanide anion [7,8]. While hundreds of examples have been reported to this day, the inherent instability of trimethylsilyl cyanohydrins sometimes limits the applications of this reaction. Therefore, alternative synthetic procedures for the preparation of stable *O*-protected cyanohydrins instead of trimethylsilyl cyanohydrin have been developed. Cyanohydrin carbonates and cyanohydrin acylates are configurationally stable and less prone to hydrolysis than cyanohydrin trimethylsilyl ethers. Therefore, one-pot synthesis of cyanohydrin carbonates and acylated cyanation using cyanoformates and cyanoacylates have recently been developed. Several catalytic reactions have been recently studied. To date, reactions using tributyltin cyanide [9], K_2_CO_3_ [10], tertiary amines [11,12], DABCO (1,4-diazabicyclo[2.2.2]octane) [13,14], dimethyl sulfoxide [15], TTMPP (tris(2,4,6-trimethoxyphenyl)phsphine) [16], DMAP (*N*,*N*-dimethyl-4-aminopyridine) [17], and *N*-heterocyclic carbenes [18] as catalysts or activators have been reported. Asymmetric catalytic reactions have also been reported using cinchona alkaloids [19,20,21,22,23,24,25], Y complexes [26,27,28], Al complexes [29,30], Ti complexes [31,32,33,34,35,36,37,38,39,40], and V complexes [41]. To further extend the scope of this versatile synthetic reaction, we have now examined the use of 1,5,7-triazabicyclo[4.4.0]dec-5-ene (TBD) as a Lewis base catalyst.

It is well-known that TBD is a strong Lewis base owing to its distorted bicyclic guanidine structure [42,43]. Employing this property, multiple unique reactions have been reported using TBD as an organobase catalyst including Henry reactions [44], Wittig and Horner-Wadsworth-Emmons reactions [45], Michael reactions [46,47], intramolecular aldol reactions [48,49], ring-opening polymerization [50,51], aminolysis of esters [52,53], conjugate addition to activated alkenes [54], synthesis of pyrazolines [55], and others [56,57]. Although TBD usually behaves as a base, it also contains an acidic N–H group; therefore, TBD may act as a bifunctional acid-base catalyst [58,59,60]. Recently, we have reported the trifluoromethylation of aldehydes [61] and the ring-opening reactions of aziridines [62,63] catalyzed using TBD as an organocatalyst. Here, we report that TBD acts as an effective organobase catalyst for the cyanation of carbonyl compounds using methyl cyanoformate or acetyl cyanide as cyanide source.

## 2. Results and Discussion

Initially, the cyanomethoxycarbonylation reaction of *p*-anisaldehyde using methyl cyanoformate was examined in the presence of 5 mol % of TBD in THF at room temperature. The desired product was obtained in 90% yield after 2 h (Table 1, entry 1). The product was obtained in a lower yield when DMF (*N*,*N*-dimethylformamide), toluene or ClCH_2_CH_2_Cl was used as the solvent. An approximately comparable yield was observed when CH_3_CN was used (Table 1, entry 2). The yield was decreased when 1 mol % of TBD was used (Table 1, entry 6). To clarify the scope of this reaction, the cyanation of several aldehydes was examined in the presence of 5 mol % of TBD. In the case of aromatic aldehydes, the yield was influenced by the substituents on the aromatic ring. With aromatic aldehydes bearing an electron-withdrawing group, it was necessary to change the order of reagent addition to obtain the desired product in good yield. Using other aromatic aldehydes, the products were obtained in good to high yields. *trans*-Cinnamaldehyde was subjected to our reaction conditions, and the 1,2-addition product was obtained in 85% yield. Although slightly longer reaction times were required, aliphatic aldehydes also produced similar high yields.

Ketones were also examined using the 10 mol % of TBD (Table 2). In the case of aliphatic ketones, good results were obtained for both acyclic and cyclic ketones. The reaction proceeded more smoothly than the reported example [17]. Only 1,2-addition products were observed for unsaturated ketones. On the other hand, for aromatic ketones, only a small amount of the desired product was obtained at room temperature even after 24 h. When the reactions were performed at elevated temperature (50 °C), a variety of aromatic ketones were converted to their corresponding cyanohydrin carbonates in moderate to good yields. In this reaction, novel cyanohydrin carbonates (**4c**, **4g**) could be obtained. In addition, **4d** and **4e** were obtained for the first time as products of this one-pot method. Unfortunately, in the reaction of 4-methoxyacetophenone, no product was found under these conditions.

Acetyl cyanide was also examined in the TBD-catalyzed reaction (Table 3). The acylated cyanohydrin products were readily obtained in high yields in a similar manner for aromatic aldehydes. Although the bond between the acetyl and cyanide groups is stronger in acetyl cyanide than in methyl cyanoformate, the reactivity did not decrease. In the case of 4-nitrobenzaldehyde, the starting material was rapidly consumed; however, the product yield was not good. In this case, it is likely that attack at the NO_2_ group occurred. Longer reaction times were needed in the reaction of aromatic aldehydes bearing electron-donating groups on the aromatic ring. This trend has been similarly reported in a recent related report [18]. The aliphatic, heterocyclic, and α,β-unsaturated aldehydes also reacted to give the corresponding products in similarly high yields.

Furthermore, we applied a polymer-supported TBD, PS-TBD [64,65,66,67] to this reaction (Table 4). Polymer-supported catalysts have attracted significant attention in recent decades because of their inherent advantages in synthetic chemistry, e.g., the simplification of reaction procedures including the easy recovery of the catalyst using filtration, application to automated systems, and recycling of the catalyst [68,69,70,71,72,73]. Thus, we examined the cyanomethoxycarbonylation reaction of *p*-anisaldehydes in the presence of 10 mol % of PS-TBD [63,74,75,76,77,78,79]. The reaction proceeded smoothly and the desired product was obtained in 95% yield after 3 h at room temperature. A variety of aldehydes reacted well with methyl cyanoformate to give cyanohydrin carbonates. Aromatic aldehydes with either electron-donating or electron-withdrawing groups afforded the corresponding products in high yields. Aliphatic aldehydes also worked well. In the reaction with aldehyde, the catalytic reactivity of PS-TBD seems to be slightly lower than TBD. Ketones were also examined; however, the reaction was very slow. Then, the reaction was performed at elevated temperature. The desired product was obtained at moderate to yield in 4 h at 50 °C. In addition, the recovery and reuse of PS-TBD for the reaction of *p*-anisaldehyde with methyl cyanoformate was also examined. After the reaction was completed, ethyl acetate was added to the reaction mixture and the catalyst was recovered by filtration. The recovered catalyst was washed, dried and then reused. The catalyst was reused, maintaining its catalytic activity after three times.

**Scheme 1:**
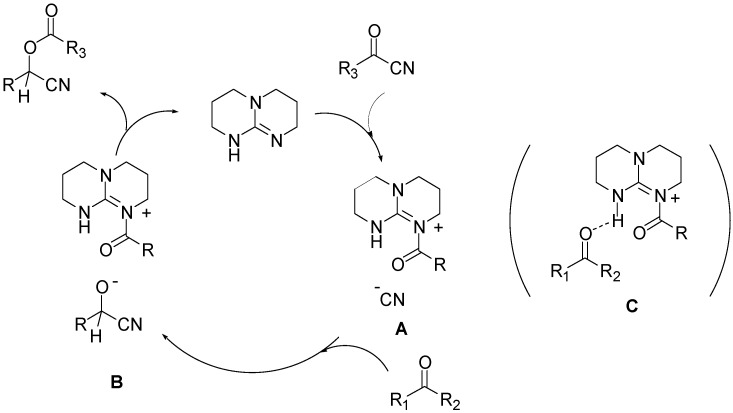
Proposed mechanism. [N⁺C(O)R]CN⁻ intermediate **A**; alkoxide anion of the cyanohydrin **B** and alternative transition state **C**.

A possible mechanism for the TBD-catalyzed cyanation is illustrated in Scheme 1. First, the nitrogen atom of TBD activates the cyanoformates or cyanoacylates to form [N⁺C(O)R]CN⁻ intermediate **A**. Next, the CN⁻ anion immediately reacts with the carbonyl compounds to give the alkoxide anion of the cyanohydrin **B**. Finally, the alkoxide anion is carbonylated or acylated to give the *O*-protected cyanohydrin with the regeneration of TBD. In this reaction, acidic N–H group of TBD may activate the carbonyl group (**C**).

## 3. Experimental Section

### 3.1. General

IR spectra were recorded on a JUSCO FT/IR-430 spectrometer (JASCO Corporation, Tokyo, Japan). ^1^H-NMR spectra were determined for solutions in CDCl_3_ with Me_4_Si as internal standard (CDCl_3_: 7.24 ppm, Me_4_Si: 0.00 ppm) on a Bruker Avance III instrument (Bruker Corporation, Billerica, MA, USA). ^13^C-NMR spectra were determined for solutions in CDCl_3_ with Me_4_Si as internal standard (CDCl_3_: 77.00 ppm, Me_4_Si: 0.00 ppm) on a Bruker Avance III instrument (Bruker Corporation). HRMS data were measured on a JEOL JMS-700 mass spectrometer (JEOL Ltd., Tokyo, Japan).

All reactions were performed under an argon atmosphere using oven-dried glassware. Flash column chromatography was performed using silica gel Wakogel C-200 (Wako Chemical, Osaka, Japan). Dehydrate DMF, THF, toluene and CH_3_CN were purchased from Kanto Kagaku (Tokyo, Japan) as the “anhydrous” and stored over 4A molecular sieves under Ar. Other commercially available reagent was used as received without further purification.

### 3.2. Method

#### 3.2.1. General Procedure for TBD-Catalyzed Cyanation Reactions of Carbonyl Compounds with Methyl Cyanoformate

TBD was dried in vacuo before use. To a solution of TBD (0.05 mmol) in CH_3_CN (1 mL) was added carbonyl compound (1.0 mmol) and methyl cyanoformate (1.25 mmol) at room temperature. After the reaction was complete (as determined by TLC), the reaction mixture was quenched with water. The resultant mixture was extracted with EtOAc (3 × 10 mL). The combined organic layers were washed with water and brine and dried with Na_2_SO_4_. After the filtration, the residue was concentrated in vacuo. The crude product was purified by column chromatography on silica gel (EtOAc:hexane = 1:5 → 1:3) to give the corresponding product.

#### 3.2.2. General Procedure for TBD-Catalyzed Cyanation Reactions of Aldehydes with Acetyl Cyanide

TBD was dried in vacuo before use. To a solution of TBD (0.10 mmol) in CH_3_CN (1 mL) was added aldehyde (1.0 mmol) and acetyl cyanide (1.5 mmol) at room temperature. After the reaction was complete (as determined by TLC), the reaction mixture was quenched with water. The resultant mixture was extracted with EtOAc (3 × 10 mL). The combined organic layers were washed with water and brine and dried with Na_2_SO_4_. After the filtration, the residue was concentrated in vacuo. The crude product was purified by column chromatography on silica gel (EtOAc:hexane = 1:10 → 1:5) to give the corresponding product.

#### 3.2.3. General Procedure for PS-TBD Catalyzed Cyanation Reactions of Carbonyl Compounds with Methyl Cyanoformate

PS-TBD was dried in vacuo before use. To a solution of PS-TBD (0.10 mmol) in CH_3_CN (1 mL) was added carbonyl compound (1.0 mmol) and methyl cyanoformate (1.25 mmol) at room temperature. After the reaction was complete (as determined by TLC), EtOAc (10 mL) was added to the mixture and PS-TBD was separated by filtration. The filtrate was washed with water and brine and dried with Na_2_SO_4_. After the filtration, the residue was concentrated in vacuo. The crude product was purified by column chromatography on silica gel (EtOAc:hexane = 1:5 → 1:3) to give the corresponding product. The recovered catalyst is reusable after washing (acetone and water) and drying in vacuo.

### 3.3. General Characterization of the Products

*Cyano(4-methoxyphenyl)methyl methyl carbonate* (**2a**) [11]: IR (neat): 2245, 1761 cm⁻^1^; ^1^H-NMR (500 MHz, CDCl_3_): δ 3.80 (s, 3H), 3.82, (s, 3H), 6.18 (s, 1H), 6.92 (d, *J* = 8.8 Hz, 2H), 7.44 (d, *J* = 8.8 Hz, 2H); ^13^C-NMR (125 MHz, CDCl_3_): δ 55.5, 55.7, 66.4, 114.6, 115.9, 123.2, 129.8, 154.1, 161.4. The corresponding spectra are shown in Supplementary Materials.

*Cyano(phenyl)methyl methyl carbonate* (**2b**) [11]: IR (neat): 2249, 1760 cm⁻^1^; ^1^H-NMR (500 MHz, CDCl_3_): δ 3.77 (s, 3H), 6.18 (s, 1H), 7.35–7.46 (m, 5H); ^13^C-NMR (125 MHz, CDCl_3_): δ 55.9, 66.6, 115.7, 127.9, 129.3, 130.7, 131.1, 154.1.

*Cyano(4-chlorophenyl)methyl methyl carbonate* (**2c**) [11]: IR (neat): 2250, 1758 cm⁻^1^; 2347, 1762 cm⁻^1^; ^1^H-NMR (500 MHz, CDCl_3_): δ 3.84 (s, 3H), 6.21 (s, 1H), 7.40 (d, *J* = 8.6 Hz, 2H), 7.46 (d, *J* = 8.6 Hz, 2H); ^13^C-NMR (125 MHz, CDCl_3_): δ 56.0, 65.8, 115.3, 129.2, 129.3, 129.6, 136.9, 153.9.

*Cyano(4-nitrophenyl)methyl methyl carbonate* (**2d**) [16]: IR (neat): 2251, 1760 cm⁻^1^; ^1^H-NMR (500 MHz, CDCl_3_): δ 2.20 (s, 3H), 6.49 (s, 1H), 7.71 (d, *J* = 8.7 Hz, 2H), 8.30 (d, *J* = 8.7 Hz, 2H); ^13^C-NMR (125 MHz, CDCl_3_): 20.3, 61.6, 115.1, 124.4, 128.7, 138.1, 149.0, 168.6.

*Cyano(naphthalen-1-yl)methyl methyl carbonate* (**2e**) [15]: IR (neat): 2247, 1768 cm⁻^1^; ^1^H-NMR (500 MHz, CDCl_3_): δ 3.77 (s, 3H), 6.18 (s, 1H), 7.51–7.58 (m, 3H), 7.80–7.96 (m, 4H); ^13^C-NMR (125 MHz, CDCl_3_): δ 56.0, 65.3, 115.8, 122.6, 125.0, 126.4, 127.7, 129.1, 129.2, 130.0, 131.8, 133.9, 154.2.

*Cyano(naphthalen-2-yl)methyl methyl carbonate* (**2f**) [15]: IR (neat): 2254, 1757 cm⁻^1^; ^1^H-NMR (500 MHz, CDCl_3_): δ 3.85 (s, 3H), 6.42 (s, 1H), 7.51–7.56 (m, 3H), 7.84–7.96 (m, 4H); ^13^C-NMR (125 MHz, CDCl_3_): δ 55.9, 66.8, 115.8, 124.1, 127.1, 127.7, 127.9, 128.1, 128.4, 128.5, 129.5, 132.8, 134.0, 154.2.

*Cyano-3-phenylpropen-2-yl methyl carbonate* (**2g**) [15]: IR (neat): 2260, 1759 cm^−1^; ^1^H-NMR (500 MHz, CDCl_3_): δ 3.86 (s, 3H), 5.88(dd, *J* = 1.1, 6.8 Hz, 1H), 6.20(dd, *J* = 6.8, 15.8 Hz, 1H), 6.98 (d, *J* = 15.8 Hz, 1H), 7.33–7.38 (m, 3H), 7.40–7.46 (m, 2H); ^13^C-NMR (125 MHz, CDCl_3_): δ 55.9, 65.2, 114.9, 117.6, 127.2, 128.2, 129.5, 134.2, 138.4, 154.0.

*Cyano-3-phenylpropyl methyl carbonate* (**2h**) [15]: IR (neat): 2240, 1760 cm⁻^1^; ^1^H-NMR (500 MHz, CDCl_3_): δ 2.22–2.30 (m, 2H), 2.85 (t, *J* = 7.8 Hz, 2H), 3.86 (s, 3H), 5.13 (t, *J* = 7.8 Hz, 2H), 7.18–7.21 (m, 3H), 7.29–7.33 (m, 2H); ^13^C-NMR (125 MHz, CDCl_3_): δ 30.4, 33.8, 55.8, 64.0, 116.2, 126.7, 128.2, 128.4, 128.7, 129.0, 138.7, 154.0.

*Cyano(cyclohexyl)methyl methyl carbonate* (**2i**) [15]: IR (neat): 2247, 1758 cm⁻^1^; ^1^H-NMR (500 MHz, CDCl_3_): δ 1.06–1.25 (m, 5H), 1.65 (d, *J* = 12.7 Hz, 1H), 1.89 (m, 6H), 3.79 (s, 3H), 4.98 (d, *J* = 6.0 Hz, 1H) 3.80 (s, 3H), 6.28 (s, 1H), 6.37 (dd, *J* = 1.9, 3.4 Hz, 1H), 6.66 (d, *J* = 3.4 Hz, 1H), 7.45 (m, 1H); ^13^C-NMR (125 MHz, CDCl_3_): δ 25.2, 27.7, 27.9, 40.1, 55.7, 69.5, 115.7, 154.3.

*Cyano(1-octyl)methyl methyl carbonate* (**2j**) [16]: IR (neat): 2249, 1760 cm⁻^1^; ^1^H-NMR (500 MHz, CDCl_3_): δ 0.81 (m, 3H), 1.19–1.21 (m, 12H), 1.87 (dd, *J* = 6.8, 15.8 Hz, 2H), 3.79 (s, 3H), 5.13 (t, *J* = 6.8 Hz, 1H); ^13^C-NMR (125 MHz, CDCl_3_): δ 14.0, 22.6, 24.4, 28.8, 29.2, 29.5, 31.7, 32.3, 55.7, 64.9, 116.5, 154.3.

*Cyano(furan-2-yl)methyl methyl carbonate* (**2k**) [11]: IR (neat): 2247, 1768 cm⁻^1^; ^1^H-NMR (500 MHz, CDCl_3_): δ 3.80 (s, 3H), 6.28 (s, 1H), 6.37 (dd, *J* = 1.9, 3.4 Hz, 1H), 6.66 (d, *J* = 3.4 Hz, 1H), 7.45 (m, 1H); ^13^C-NMR (125 MHz, CDCl_3_): δ 56.0, 59.3, 111.2, 113.2, 143.5, 145.4, 153.9.

*1-Cyano-1-methyl-3-phenylpropyl methyl carbonate* (**4a**) [11]: IR (neat): 2250, 1760 cm⁻^1^; ^1^H-NMR (500 MHz, CDCl_3_): δ 1.82 (s, 3H), 2.17–2.34 (m, 2H), 2.82–2.90 (m, 2H), 3.83 (s, 3H), 7.16–7.24 (m, 3H), 7.26–7.31 (m, 2H); ^13^C-NMR (125 MHz, CDCl_3_): δ 24.5, 30.2, 41.6, 55.3, 74.0, 118.2, 126.5, 128.4, 128.7, 139.7, 153.2.

*1-Cyano-1-ethylhexyl methyl carbonate* (**4b**) [16]: IR (neat): 2250, 1759 cm⁻^1^; ^1^H-NMR (500 MHz, CDCl_3_): δ 0.85 (t, *J* = 6.9 Hz, 3H), 1.03 (t, *J* = 7.5 Hz, 3H), 1.24–1.33 (m, 4H), 1.35–1.54 (m, 2H), 1.88–2.10 (m, 4H), 3.78 (s, 3H); ^13^C-NMR (125 MHz, CDCl_3_): δ 7.9, 13.8, 22.3, 23.2, 29.8, 31.3, 36.0, 55.1, 78.5, 117.8, 153.2.

*1-Cyanocyclohexyl methyl carbonate* (**4c**): colorless oil; IR (neat): 2250, 1760 cm⁻^1^; ^1^H-NMR ( 500 MHz, CDCl_3_ ) : δ 1.15–1.35 (m, 1H), 1.53–1.64 (m, 3H), 1.70–1.77 (m, 2H), 1.80–1.85 (m, 2H), 2.26–2.28 (m, 2H), 3.79 (s, 3H); ^13^C-NMR (125 MHz, CDCl_3_): δ 22.0, 24.3, 35.0, 55.1, 75.0, 118.2, 153.0; HRMS (FAB): *m*/*z*: calcd for C_9_H_14_NO_3_: 184.0973; found: 184.0991 [M + H]^+^.

*1-cyanocyclohex-2-en-1-yl methyl carbonate* (**4d**) [80]: IR (neat): 2245, 1753 cm⁻^1^; ^1^H-NMR (500 MHz, CDCl_3_ ): δ 1.68–72 (m, 2H), 1.97–1.99 (m, 2H), 2.40 (t, *J* = 6.6 Hz, 2H), 3.81 (s, 3H), 5.98 (d, *J* = 10 Hz, 1H), 6.99 (ddd, *J* = 7.1, 7.1, 4.1 Hz, 1H); ^13^C-NMR (125 MHz, CDCl_3_ ): δ 20.9, 22.7, 24.3, 51.9, 74.4, 112.5, 116.9, 129.8, 152.9.

*1-Cyano-1-methyl-3-phenylprope-2-yl methyl carbonate* (**4e**) [81]: IR (neat): 2250, 1760 cm⁻^1^; ^1^H-NMR (500 MHz, CDCl_3_) : δ 1.84 (s, 3H), 3.72 (s, 3H), 6.10 (d, *J* = 16.0 Hz, 1H), 6.95 (d, *J* = 16.0 Hz, 1H), 7.29–7.30 (m, 3H), 7.43–7.44 (m, 2H); ^13^C-NMR (125 MHz, CDCl_3_ ): δ 14.2, 21.1, 55.4, 60.4, 73.8, 117.3, 124.0, 128.8, 130.5, 134.4, 152.9.

*1-Cyano-1-phenylethyl methyl carbonate* (**4f**) [11]: IR (neat) 2250, 1752 cm⁻^1^; ^1^H-NMR (500 MHz, CDCl_3_): δ 2.01 (s, 3H), 3.77 (s, 3H), 7.37–7.46 (m, 3H), 7.53–7.55 (m, 2H); ^13^C-NMR (125 MHz, CDCl_3_): δ 29.9, 55.4, 75.7, 117.9, 124.5, 129.1, 129.5, 137.6, 152.9.

*1-Cyano-1-phenylpropyl methyl carbonate* (**4g**): pale yellow oil; IR (neat) 2249, 1758 cm⁻^1^; ^1^H-NMR (500 MHz, CDCl_3_): δ 1.03 (t, *J* = 7.4 Hz, 3H), 2.05–2.12 (m, 1H), 2.27–2.34 (m, 1H), 3.74 (s, 3H), 7.36–7.42 (m, 5H); ^13^C-NMR (125 MHz, CDCl3): δ 8.5, 36.0, 55.4, 65.6, 80.4, 117.0, 124.8, 129.0. HRMS (FAB): *m*/*z*: calcd for C_9_H_14_NO_3_: 220. 0968; found: 220. 0979 [M + H]^+^.

*1-Cyano-1-(4-chlorophenyl)ethyl methyl carbonate* (**4h**) [11]: IR (neat) 2250, 1760 cm⁻^1^; ^1^H-NMR (500 MHz, CDCl_3_): δ 1.99 (s, 3H), 3.76 (s, 3H), 7.38 (d, *J* = 8.7 Hz, 2H), 7.48 (d, *J* = 8.7 Hz, 2H); ^13^C-NMR (125 MHz, CDCl_3_): δ 29.7, 55.5, 66.0, 75.1, 117.5, 126.1, 129.3, 135.6, 136.1, 152.8.

*1-Cyano-1-(4-nitrophenyl)ethyl methyl carbonate* (**4i**) [11]: IR (neat) 2251, 1749 cm⁻^1^; ^1^H-NMR (500 MHz, CDCl_3_): δ 2.00 (s, 3H), 3.76 (s, 3H), 7.70 (d, *J* = 8.9 Hz, 2H), 8.06 (d, *J* = 8.9 Hz, 2H); ^13^C-NMR (125 MHz, CDCl_3_): δ 29.7, 55.8, 65.8, 74.8, 117.0, 123.8, 124.4, 125.7, 152.8.

*Cyano(phenyl)methyl methyl* *acetate* (**5a**) [14]: IR (neat) 2250, 1742 cm⁻^1^; ^1^H-NMR (500 MHz, CDCl_3_): δ 2.17 (s, 3H), 6.39 (s, 1H), 7.44 (m, 3H), 7.51 (d, *J* = 11.1 Hz, 2H); ^13^C-NMR (125 MHz, CDCl_3_): δ 20.4, 62.8, 116.1, 127.9, 129.2, 130.4, 131.7, 168.9.

*Cyano(4-chlorophenyl)methyl methyl* *acetate* (**5b**) [18]: IR (neat) 2245, 1756 cm⁻^1^; ^1^H-NMR (500 MHz, CDCl_3_): δ 2.15 (s, 3H), 6.36 (s, 1H), 7.41 (d, *J* = 8.7 Hz, 2H), 7.45 (d, *J* = 8.7 Hz, 2H); ^13^C-NMR (125 MHz, CDCl_3_): δ 20.4, 62.1, 115.7, 129.3, 129.5, 130.2, 136.6, 168.8.

*Cyano(4-methylphenyl)methyl methyl* *acetate* (**5c**) [18]: IR (neat) 2250, 1753 cm⁻^1^; ^1^H-NMR (500 MHz, CDCl_3_): δ 2.13 (s, 3H), 2.37 (s, 3H), 6.35 (s, 1H), 7.23 (d, *J* = 8.0 Hz, 2H), 7.39 (d, *J* = 8.0 Hz, 2H); ^13^C-NMR (125 MHz, CDCl_3_): δ 20.5, 21.3, 62.7, 116.2, 127.9, 128.8, 129.9, 140.7, 169.0.

*Cyano(4-methoxyphenyl)methyl methyl* *acetate* (**5d**) [18]: IR (neat) 2249, 1747 cm⁻^1^; ^1^H-NMR (500 MHz, CDCl_3_): δ 2.12 (s, 3H), 3.81 (s, 3H), 6.34 (s, 1H), 6.93 (d, *J* = 8.8 Hz, 2H), 7.43 (d, *J* = 8.8 Hz, 2H); ^13^C-NMR (125 MHz, CDCl_3_): δ 20.5, 55.4, 62.6, 114.5, 116.3, 123.8, 129.6, 161.1, 169.0.

*Cyano(4-nitrophenyl)methyl methyl* *acetate* (**5e**) [82]: IR (neat): 2251, 1760 cm⁻^1^; ^1^H-NMR (500 MHz, CDCl_3_): δ 2.20 (s, 3H), 6.49 (s, 1H), 7.71 (d, *J* = 8.7 Hz, 2H), 8.30 (d, *J* = 8.7 Hz, 2H); ^13^C-NMR (125 MHz, CDCl_3_): δ 20.3, 61.6, 115.1, 124.4, 128.7, 138.1, 149.0, 168.6.

*Cyano(naphthalen-1-yl)methyl methyl* *acetate* (**5f**) [18]: IR (neat) 2249, 1755 cm⁻^1^; ^1^H-NMR (500 MHz, CDCl_3_): δ 2.17 (s, 3H), 7.02 (s,1H), 7.50 (dd, *J* = 7.1, 8.1 Hz, 1H), 7.54 (t, *J* = 8.1 Hz, 1H), 7.60 (t, *J* = 6.2 Hz, 1H), 7.71 (d, *J* = 6.2 Hz, 1H), 7.86 (d, *J* = 7.1 Hz, 1H), 7.94 (d, *J* = 9.9 Hz, 1H), 7.98 (d, *J* = 8.6 Hz, 1H); ^13^C-NMR (125 MHz, CDCl_3_): δ 20.4, 61.3 116.1 122.5, 125.0, 126.6, 126.9, 127.6, 129.1, 130.0, 131.5, 133.9, 169.0.

*Cyano(naphthalen-1-yl)methyl methyl* *acetate* (**5g**) [82]: IR (neat) 2250, 1753 cm⁻^1^; ^1^H-NMR (500 MHz, CDCl_3_): δ 2.17 (s, 3H), 6.57 (s, 1H), 7.53–7.57 (m, 3H), 7.87 (t, *J* = 9.3 Hz, 2H), 7.91 (d, *J* = 8.6Hz, 1H), 8.00 (d, *J* = 1.3 Hz, 1H); ^1^^3^C-NMR (125 MHz, CDCl_3_): δ 20.6, 63.1, 116.2, 124.3, 127.1, 127.6, 127.1, 127.6, 127.9, 128.1, 128.4, 129.5, 132.9, 133.9, 169.0.

*Cyano-3-phenylpropen-2-yl methyl* *acetate* (**5h**) [32]: IR (neat) 2251, 1751 cm⁻^1^; ^1^H-NMR (500 MHz, CDCl_3_): δ 2.15 (s, 3H), 6.00 (d, *J* = 6.7 Hz, 1H), 6.18 (dd, *J* = 6.7, 15.8 Hz, 1H), 6.95 (d, *J* = 15.8 Hz, 1H), 7.31–7.37 (m, 3H), 7.41 (d, *J* = 7.9 Hz, 2H); ^13^C-NMR (125 MHz, CDCl_3_): δ 20.3, 61.4, 115.4, 118.2, 127.0, 128.7, 129.3, 134.3, 137.7, 168.8.

*Cyano-3-phenylpropeyl methyl* *acetate* (**5i**) [18]: IR (neat) 2248, 1755 cm⁻^1^; ^1^H-NMR (500 MHz, CDCl_3_): δ 2.11 (s, 3H), 2.22 (dd, *J* = 7.6, 15.0 Hz, 2H), 2.82 (t, *J* = 7.6 Hz, 2H), 5.25 (t, *J* = 6.8 Hz, 1H), 7.17 (d, *J* = 7.2 Hz, 2H), 7.23 (d, *J* = 8.1 Hz, 1H), 7.30 (t, *J* = 7.2 Hz, 2H); ^13^C-NMR (125 MHz, CDCl_3_): δ 20.3, 30.7, 33.7, 60.5, 116.7, 126.7, 128.3, 128.7, 139.0, 169.1.

*Cyano(cyclohexyl)methyl methyl* *acetate* (**5j**) [18]: IR (neat) 2248, 1755 cm⁻^1^; ^1^H-NMR (500 MHz, CDCl_3_): δ 1.07–1.21 (m, 4H), 1.21–1.30 (m, 2H), 1.66–1.69 (m, 1H), 1.73–1.80 (m, 3H), 1.84–1.92 (m, 1H), 2.13 (s, 3H), 5.15 (d, *J* = 6.6 Hz, 1H); ^13^C-NMR (125 MHz, CDCl_3_): δ 20.3, 25.3, 25.7, 28.1, 40.0, 65.6, 116.2, 169.2.

*Cyano(1-octyl)methyl methyl* *acetate* (**5k**) [83]: IR (neat) 2218, 1756 cm⁻^1^; ^1^H-NMR (500 MHz, CDCl_3_): δ 0.83 (t, *J* = 7.0 Hz, 3H), 1.15–1.33 (m, 14H), 1.57 (quin, *J* = 6.8 Hz, 2H), 1.99 (s, 3H), 4.00 (t, *J* = 6.8 Hz, 2H); ^13^C-NMR (125 MHz, CDCl_3_): δ 14.0, 20.9, 22.6, 25.8, 28.5, 29.2, 29.4, 31.8, 64.6, 117.6, 171.1.

*Cyano(furan-2-yl)methyl methyl* *acetate* (**5l**) [82]: IR (neat) 2248, 1753 cm⁻^1^; ^1^H-NMR (500 MHz, CDCl_3_): δ 2.14 (s, 3H), 6.42 (dd, *J* = 1.9, 3.4 Hz, 1H), 6.45 (s, 1H), 6.66 (d, *J* = 3.4 Hz, 1H), 7.49 (dd, *J* = 0.8, 1.9 Hz, 1H); ^13^C-NMR (125 MHz, CDCl_3_): δ 20.3, 55.7, 111.0, 112.6, 114.1, 144.1, 145.0, 168.7.

## 4. Conclusions

In conclusion, we have demonstrated the TBD-catalyzed cyanation reaction of carbonyl compounds using methyl cyanoformate or acetyl cyanide as cyanide sources. A broad range of aldehydes and ketones reacted to produce *O*-protected cyanohydrins in the presence of 5–10 mol % of TBD. Furthermore, a polymer-supported catalyst, PS-TBD, also acted as a good catalyst for this reaction. PS-TBD was easily recovered and reused with minimal loss of activity. These reactions provide a simple and convenient method for synthesis of highly functionalized α-hydroxyl-carbonyl compounds and β-amino alcohols.

## Figures and Tables

**Table 1:**
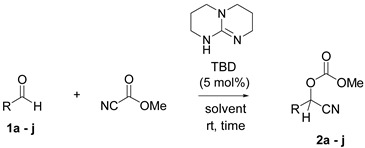
TBD (1,5,7-triazabicyclo[4,4,0]dec-5-ene) catalyzed cyanation of aldehydes with methyl cyanoformate.

Entry	Aldehyde(1)	Product	Solvent	Time	Yield (%) ^a^
1	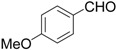	**2a**	CH_3_CN	2 h	90
2			THF	2 h	81
3			ClCH_2_CH_2_Cl	3 h	65
4			DMF	3 h	60
5			toluene	12 h	35
6 ^b^			CH_3_CN	12 h	68
7	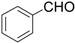	**2b**	CH_3_CN	12 h	89
8	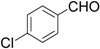	**2c**		4 h	92
9 ^c^	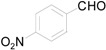	**2d**		0.5 h	70
10	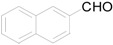	**2e**		4 h	96
11	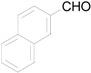	**2f**		4 h	90
12	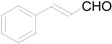	**2g**		2 h	85
13	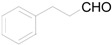	**2h**		4 h	75
14	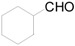	**2i**		4 h	96
15	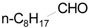	**2j**		4 h	89
16	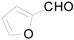	**2k**		4 h	88

^a^ Isolated yield; ^b^ 1 mol % of TBD was used; ^c^ Aldehyde was added after addition of methyl cyanoformate.

**Table 2:**
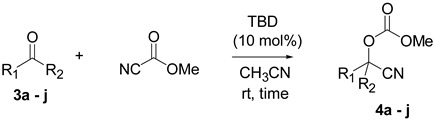
TBD catalyzed cyanation of ketones with methyl cyanoformate.

Entry	Ketone(3)	Product	Time	Yield (%) ^a^
1	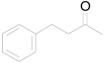	**4a**	4 h	85
2	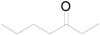	**4b**	1 h	90
3	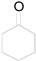	**4c**	1 h	86
4	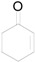	**4d**	1 h	70
5 ^b^	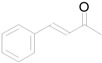	**4e**	24 h	33
6 ^b^	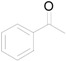	**4f**	24 h	31
7 ^b^	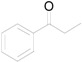	**4g**	24 h	75
8	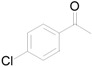	**4h**	6 h	55
9 ^b^	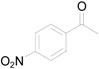	**4i**	24 h	38
10	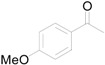	**4j**	24 h	0

^a^ Isolated yield; ^b^ at 50 °C.

**Table 3:**
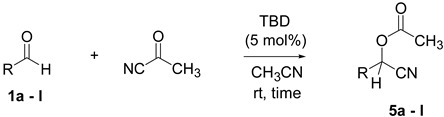
TBD catalyzed cyanation of aldehydes with acetyl cyanide.

Entry	Aldehyde(1)	Product	Time	Yield (%) ^a^
1	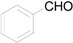	**5a**	3 h	80
2	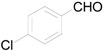	**5b**	6 h	91
3	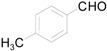	**5c**	24 h	94
4	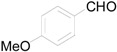	**5d**	48 h	67
5	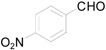	**5e**	0.5 h	40
6	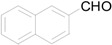	**5f**	3 h	90
7	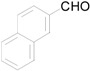	**5g**	3 h	90
8	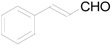	**5h**	3 h	92
9	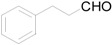	**5i**	6 h	70
10	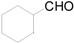	**5j**	6 h	88
11	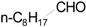	**5k**	6 h	85
12	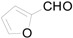	**5l**	6 h	80

^a^ Isolated yield.

**Table 4:**
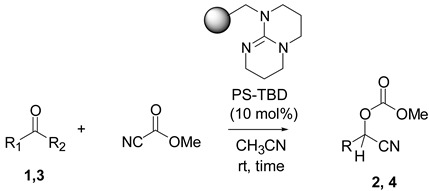
PS (polymer-supported)-TBD catalyzed cyanation of carbonyl compounds with methyl cyanoformate.

Entry	Carbonyl Compounds	Product	Time	Yield (%) ^a^
1	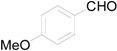	**2a**	2 h	90
2 ^b^		**2a**	2 h	88
3 ^c^		**2a**	2 h	92
4 ^d^		**2a**	2 h	88
5	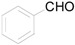	**2b**	12 h	73
6	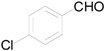	**2c**	16 h	86
7	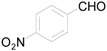	**2d**	4 h	94
8	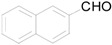	**2e**	4 h	98
9	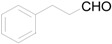	**2h**	4 h	90
10	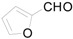	**2k**	2 h	78
11	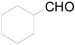	**2i**	2 h	82
12	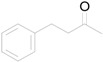	**4a**	24 h	24
13 ^e^		**4a**	12 h	75
14 ^e^	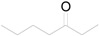	**4b**	24 h	70
15 ^e^	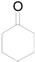	**4c**	18 h	76

^a^ Isolated yield; ^b^ 2nd run; ^c^ 3rd run; ^d^ 4th run; ^e^ at 50 °C.

## Supplementary Materials

Supplementary materials can be accessed at: http://www.mdpi.com/1420-3049/21/8/1030/s1.
