# Supplementary material for: TBD- or PS-TBD-Catalyzed One-Pot Synthesis of Cyanohydrin Carbonates and Cyanohydrin Acetates from Carbonyl Compounds
Source: Molecules. 2016 Aug 10;21(8):1030. doi: 10.3390/molecules21081030 (PMC6273017; doi:10.3390/molecules21081030)
Supplement: Supplementary file 1 [file molecules-21-01030-s001.pdf]

Supplementary Material: TBD- or PS-TBD- Catalyzed One-Pot Synthesis of Cyanohydrin Carbonates and Cyanohydrin Acetates from Carbonyl Compounds

Satoru Matsukawa, Junya Kimura and Miki Yoshioka

Instrumentation and Chemicals (S1)
Experimental procedure S1
Copies of ¹H- and ¹³C-NMR Spectra of products S2

1. Instrumentation and Chemicals

IR spectra were recorded on a JUSCO FT/IR-430 spectrometer (JASCO Corporation, Tokyo, Japan). ^{1}H-NMR spectra were determined for solutions in $CDCl_3$ with Me_4Si as internal standard ($CDCl_3$: 7.24 ppm, Me_4Si: 0.00 ppm) on a Bruker Avance III instrument (Bruker Corporation, Billerica, MA, USA). ^{13}C-NMR spectra were determined for solutions in $CDCl_3$ with Me_4Si as internal standard ($CDCl_3$: 77.00 ppm, Me_4Si: 0.00 ppm) on a Bruker Avance III instrument (Bruker Corporation). HRMS data were measured on a JEOL JMS-700 mass spectrometer (JEOL Ltd., Tokyo, Japan).

All reactions were performed under an argon atmosphere using oven-dried glassware. Flash column chromatography was performed using silica gel Wakogel C-200 (Wako Chemical, Osaka, Japan). Dehydrate DMF, THF, toluene and CH_3CN were purchased from Kanto Kagaku (Tokyo, Japan) as the "anhydrous" and stored over 4A molecular sieves under Ar. Other commercially available reagent was used as received without further purification.

2. General Procedure for TBD-Catalyzed Cyanation Reactions of Carbonyl Compounds with Methyl Cyanoformate

TBD was dried in vacuo before use. To a solution of TBD (0.05 mmol) in CH_3CN (1 mL) was added carbonyl compound (1.0 mmol) and methyl cyanoformate (1.25 mmol) at room temperature. After the reaction was complete (as determined by TLC), the reaction mixture was quenched with water. The resultant mixture was extracted with EtOAc (3 × 10 mL). The combined organic layers were washed with water and brine and dried with Na_2SO_4. After the filtration, the residue was concentrated in vacuo. The crude product was purified by column chromatography on silica gel (EtOAc:hexane = 1:5 → 1:3) to give the corresponding product.

3. General Procedure for TBD-Catalyzed Cyanation Reactions of Aldehydes with Acetyl Cyanide

TBD was dried in vacuo before use. To a solution of TBD (0.10 mmol) in CH_3CN (1 mL) was added aldehyde (1.0 mmol) and acetyl cyanide (1.5 mmol) at room temperature. After the reaction was complete (as determined by TLC), the reaction mixture was quenched with water. The resultant mixture was extracted with EtOAc (3 × 10 mL). The combined organic layers were washed with water and brine and dried with Na_2SO_4. After the filtration, the residue was concentrated in vacuo. The crude product was purified by column chromatography on silica gel (EtOAc:hexane = 1:10 → 1:5) to give the corresponding product.

4. General Procedure for PS-TBD Catalyzed Cyanation Reactions of Carbonyl Compounds with Methyl Cyanoformate

To a solution of PS-TBD (0.10 mmol) in CH_3CN (1 mL) was added carbonyl compound (1.0 mmol) and methyl cyanoformate (1.25 mmol) at room temperature. After the reaction was complete (as determined by TLC), EtOAc (10 mL) was added to the mixture and PS-TBD was separated by filtration. The filtrate was washed with water and brine and dried with Na_2SO_4. After

the filtration, the residue was concentrated in vacuo. The crude product was purified by column chromatography on silica gel (EtOAc:hexane = 1:5 → 1:3) to give the corresponding product.

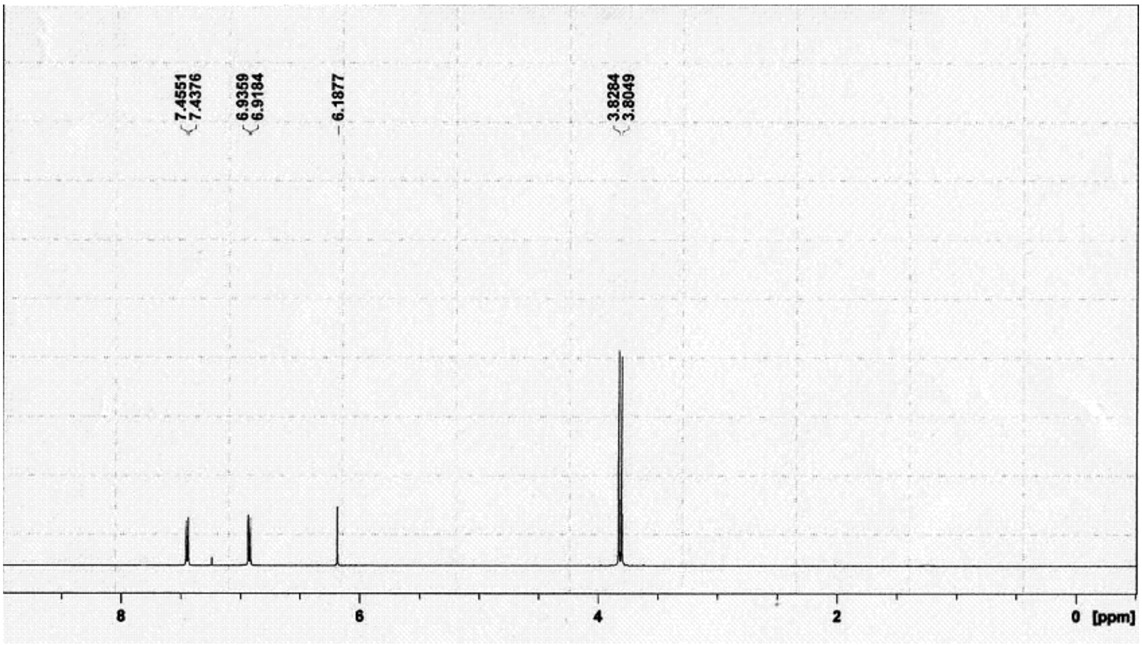

Figure S1. ^{1}H-NMR Spectrum of compound **2a**.

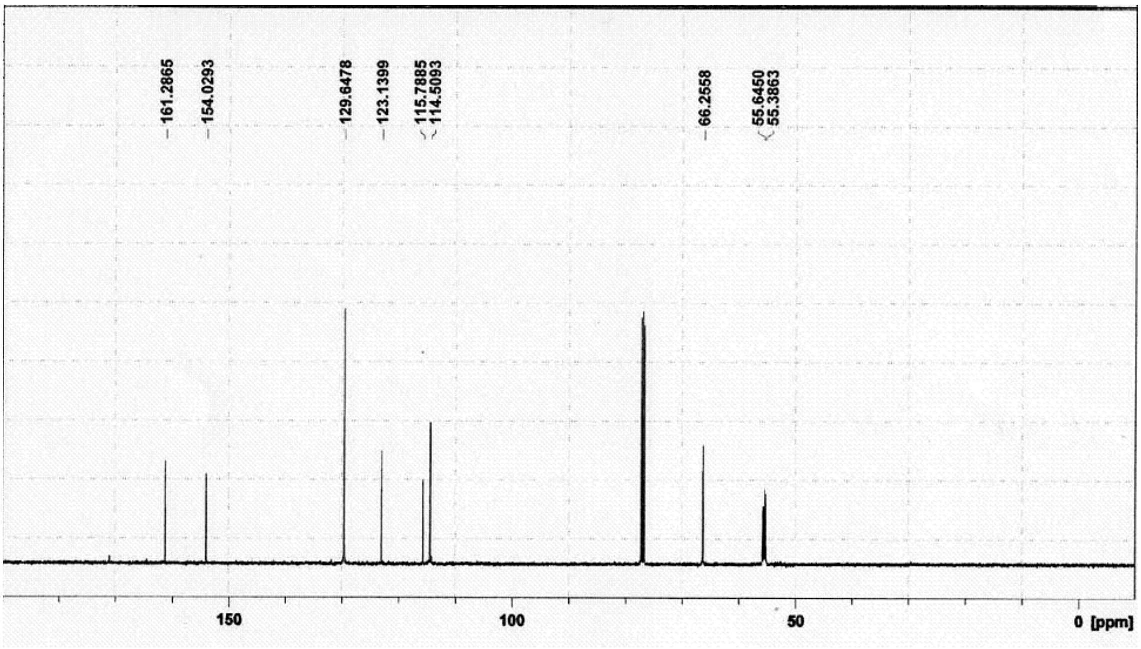

Figure S2. ^{13}C-NMR Spectrum of compound **2a**.

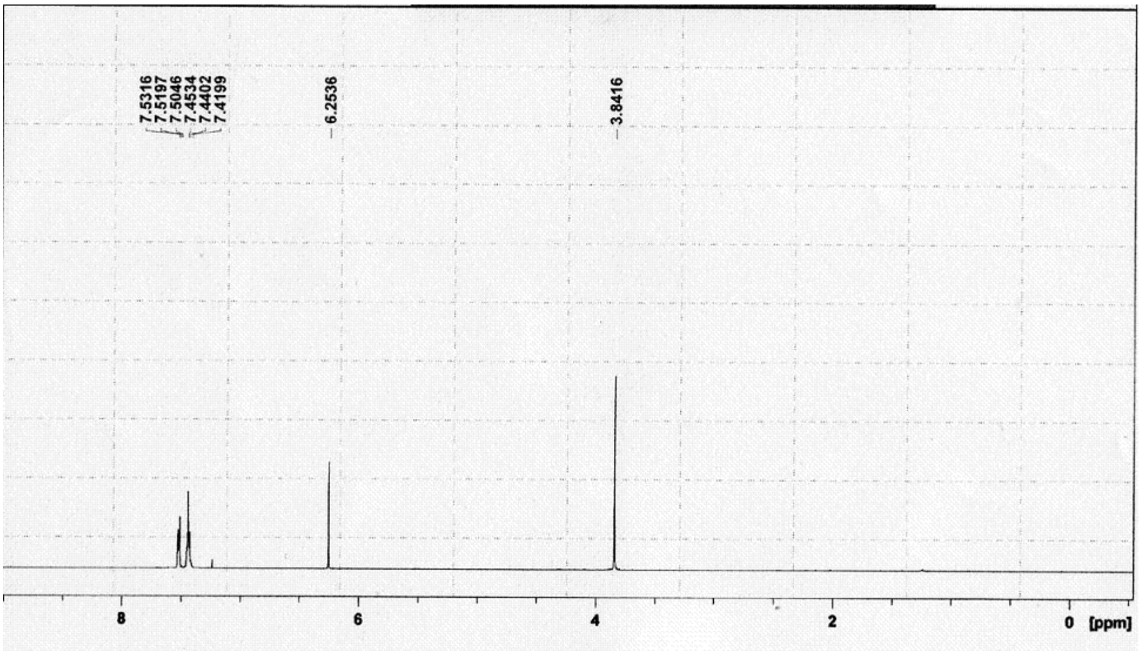

Figure S3. ^{1}H-NMR Spectrum of compound **2b**.

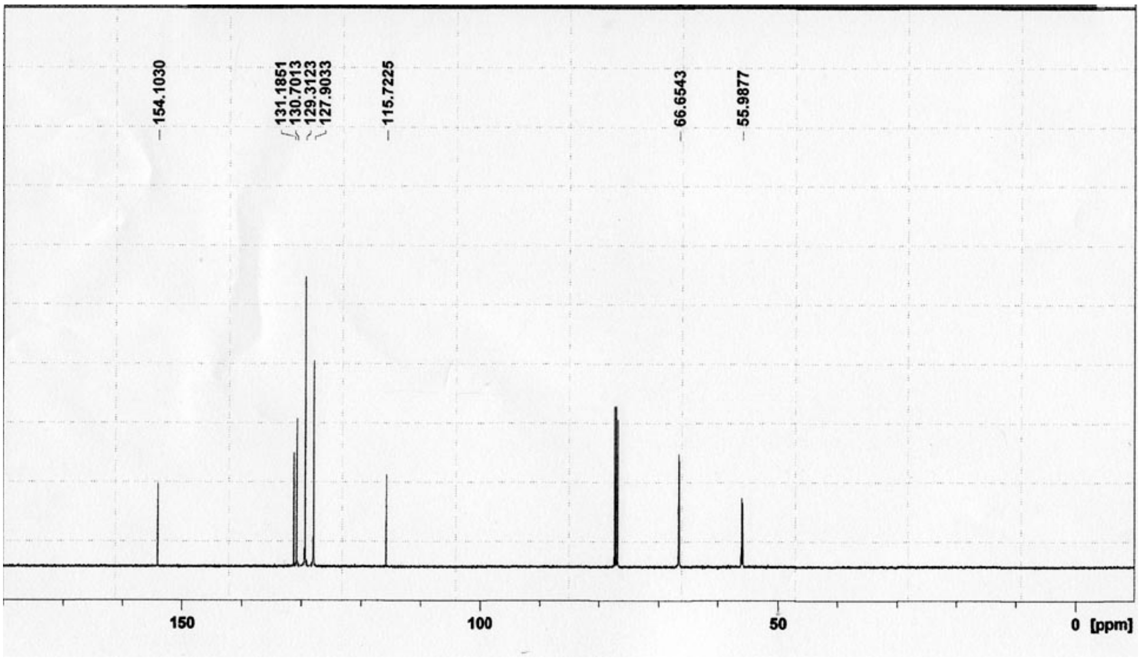

Figure S4. ^{13}C-NMR Spectrum of compound **2b**.

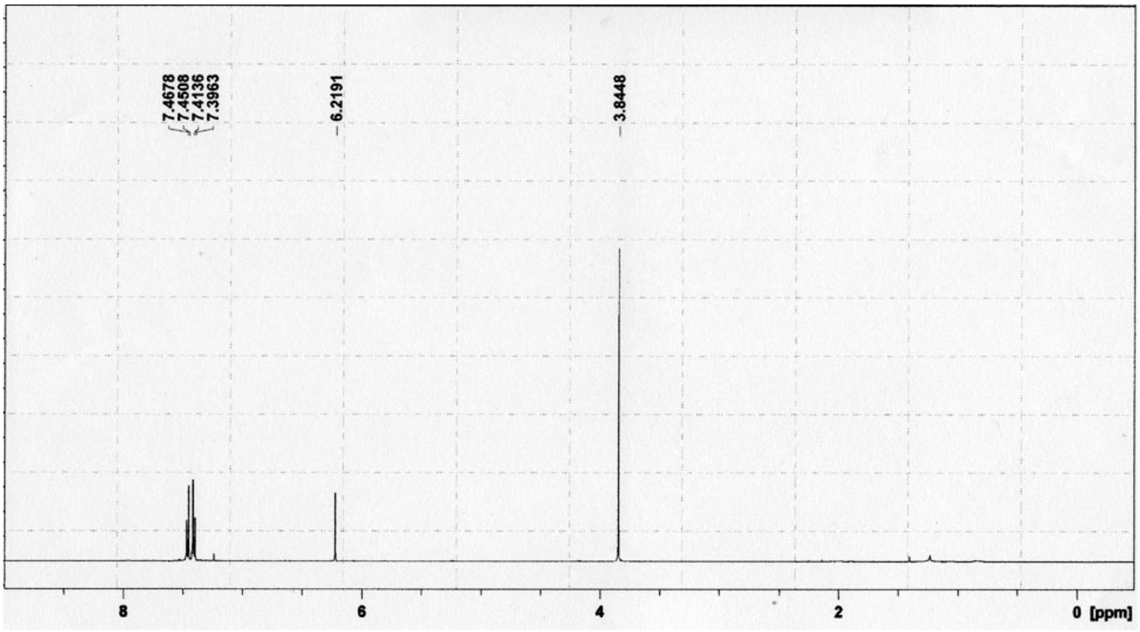

Figure S5. ^{1}H-NMR Spectrum of compound **2c**.

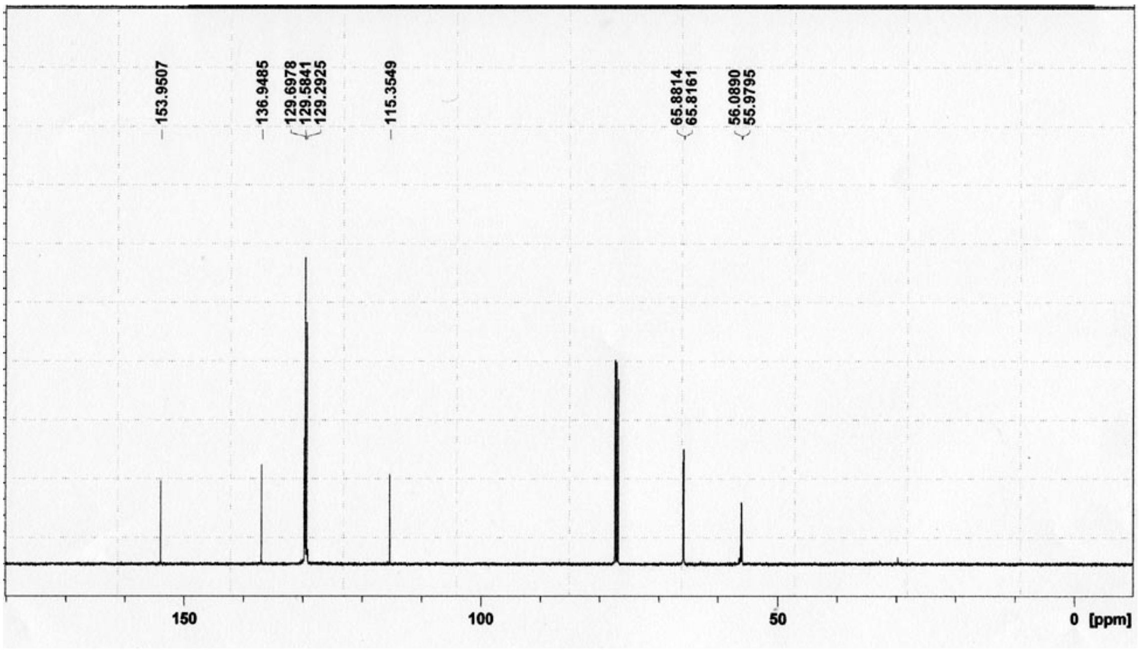

Figure S6. ^{13}C-NMR Spectrum of compound **2c**.

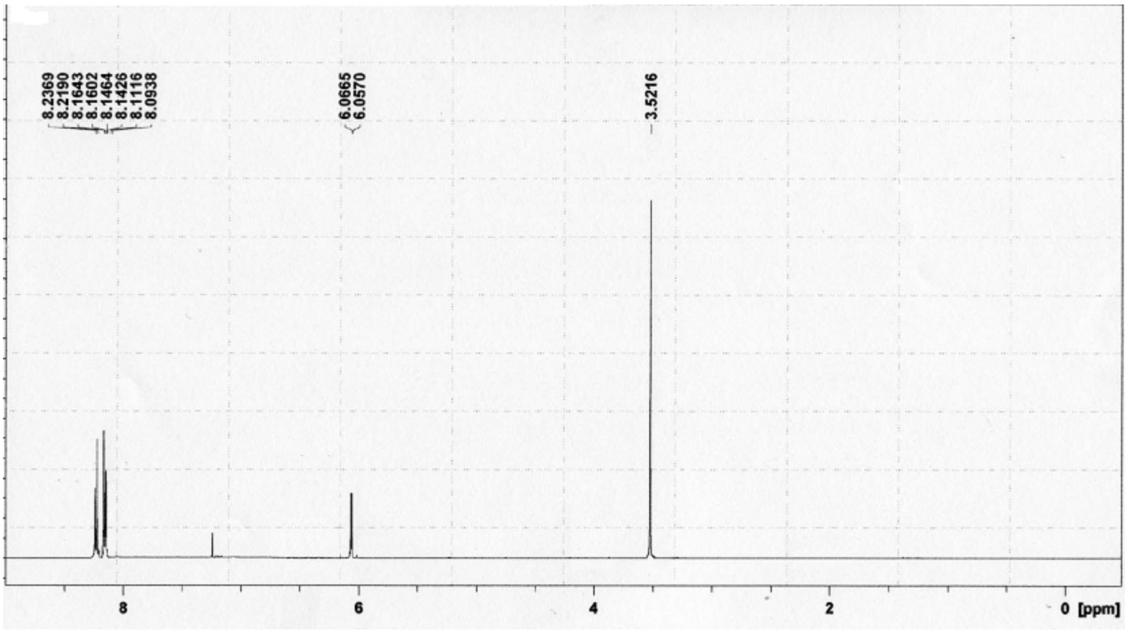

Figure S7. ^{1}H-NMR Spectrum of compound **2d**.

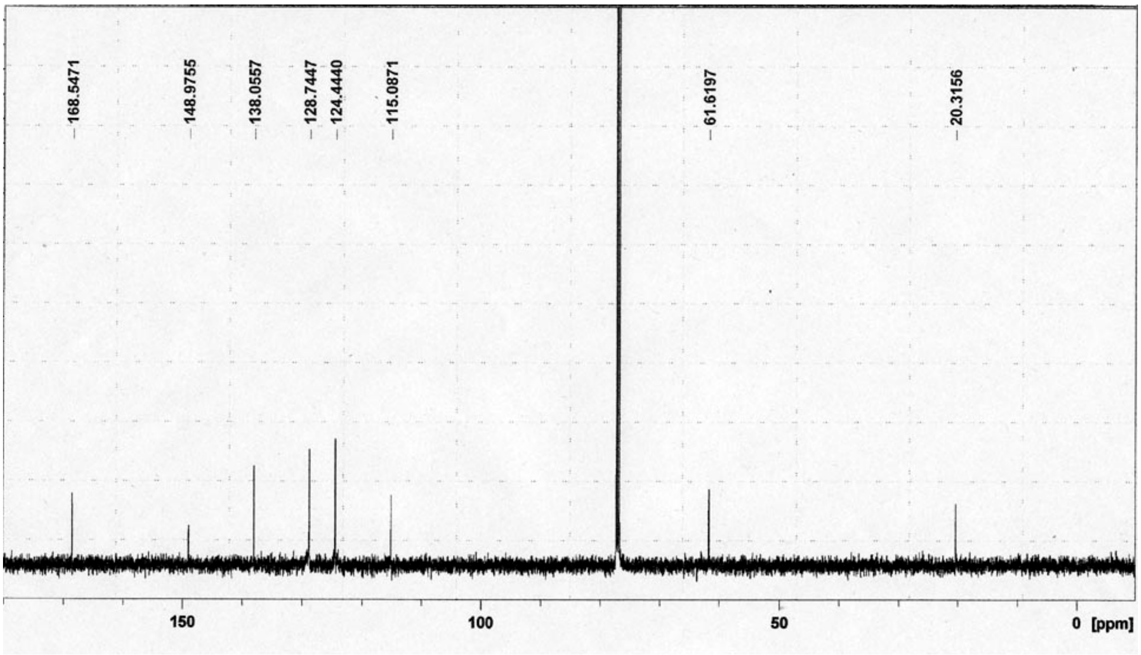

Figure S8. ^{13}C-NMR Spectrum of compound **2d**.

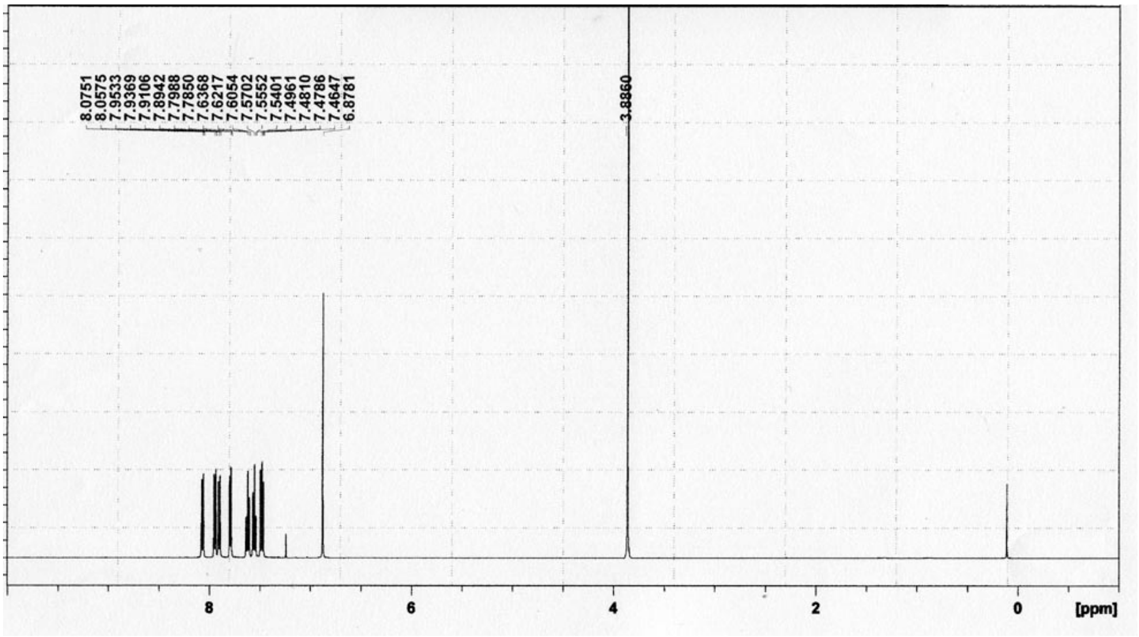

Figure S9. ^{1}H-NMR Spectrum of compound **2e**.

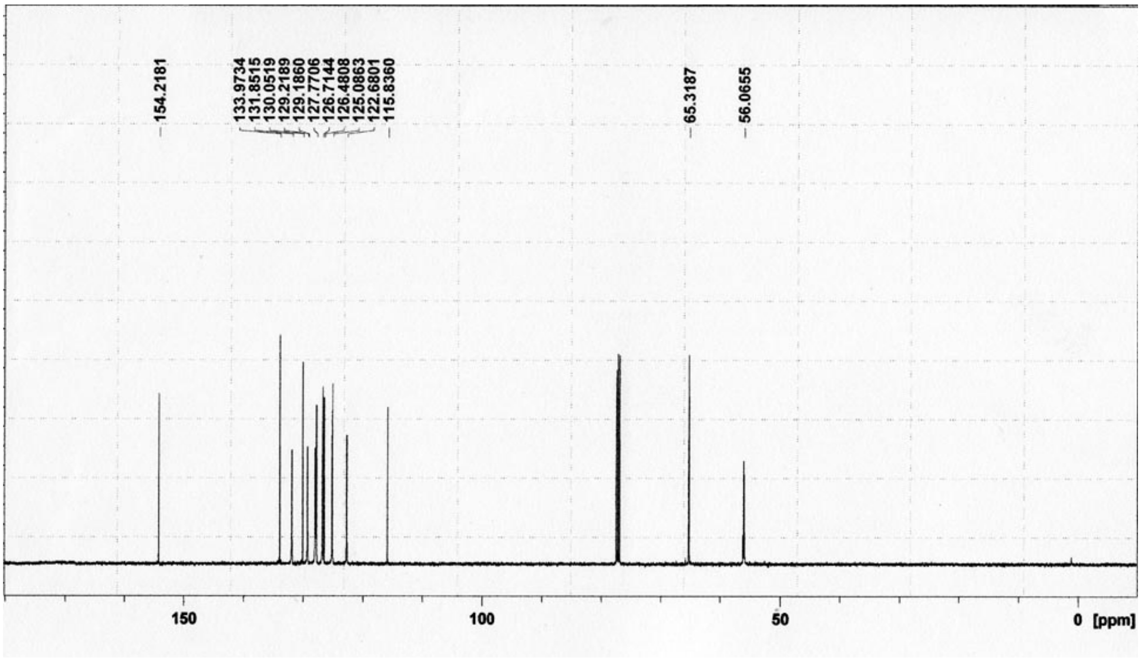

Figure S10. ^{13}C-NMR Spectrum of compound **2e**.

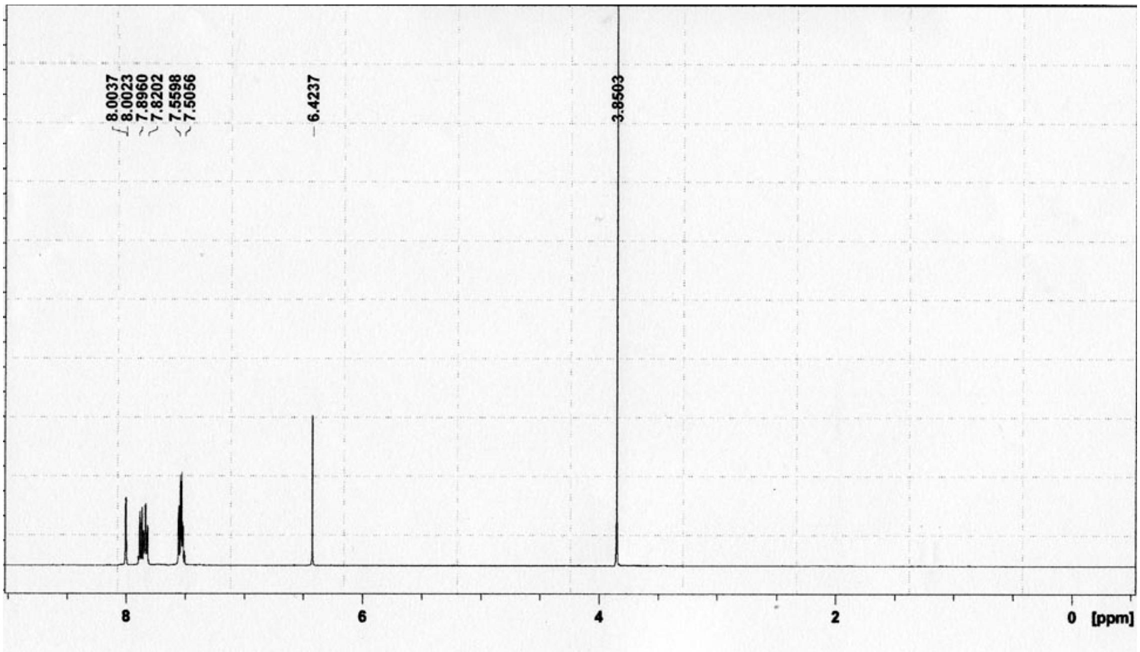

Figure S11. ^{1}H-NMR Spectrum of compound **2f**.

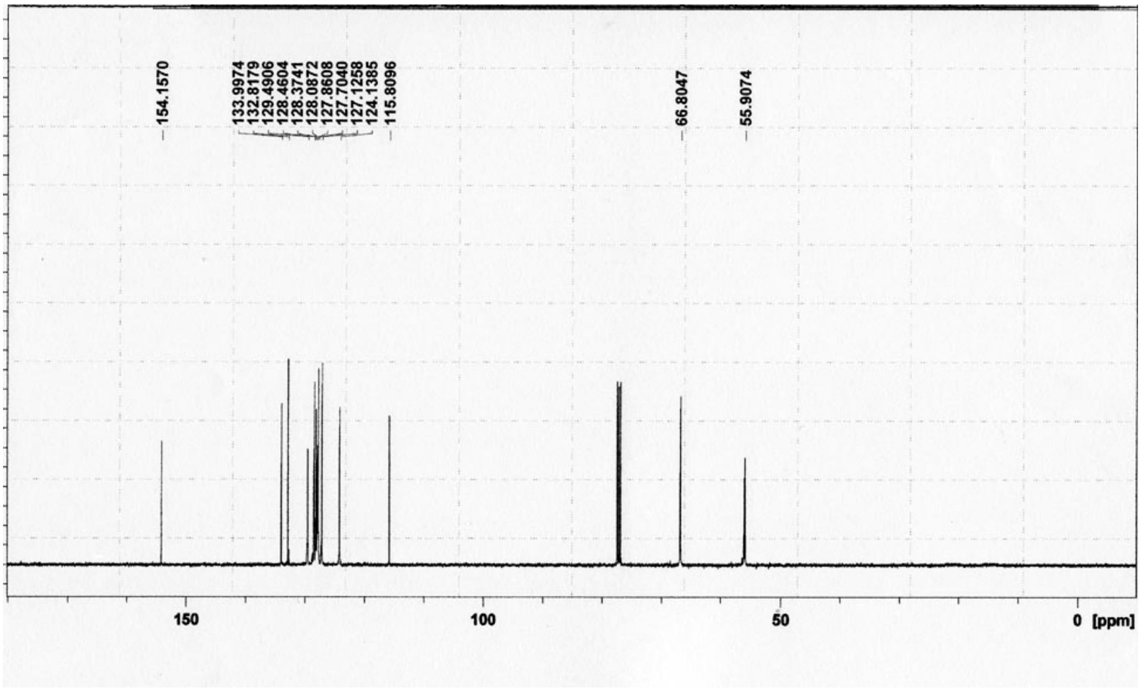

Figure S12. ^{13}C-NMR Spectrum of compound **2f**.

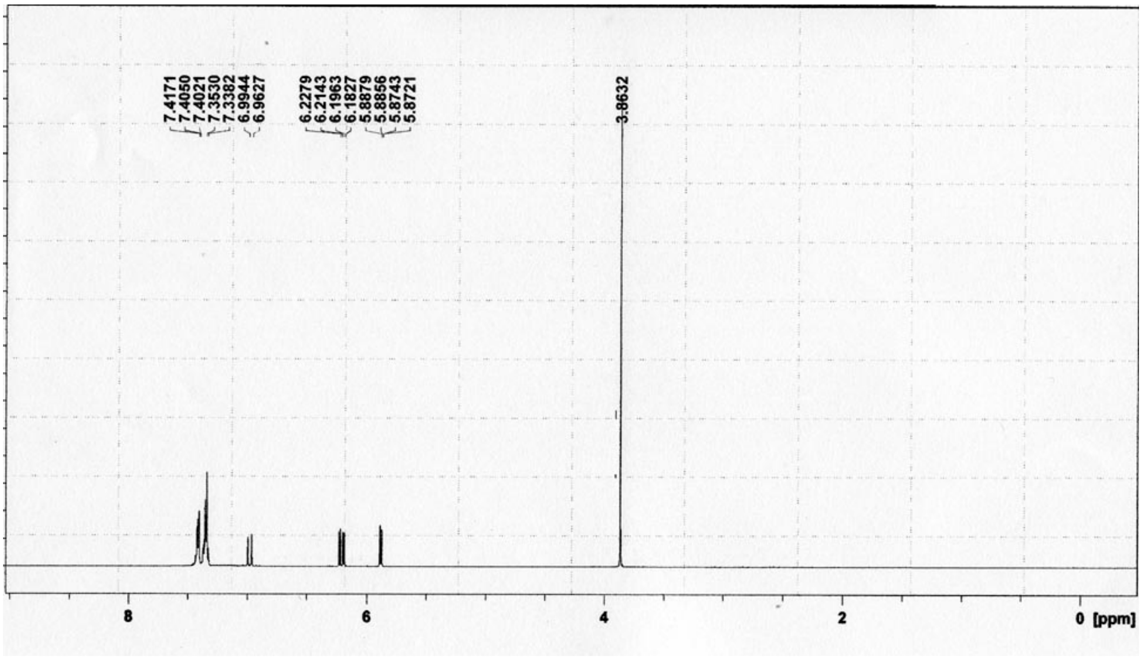

Figure S13. ^{1}H-NMR Spectrum of compound **2g**.

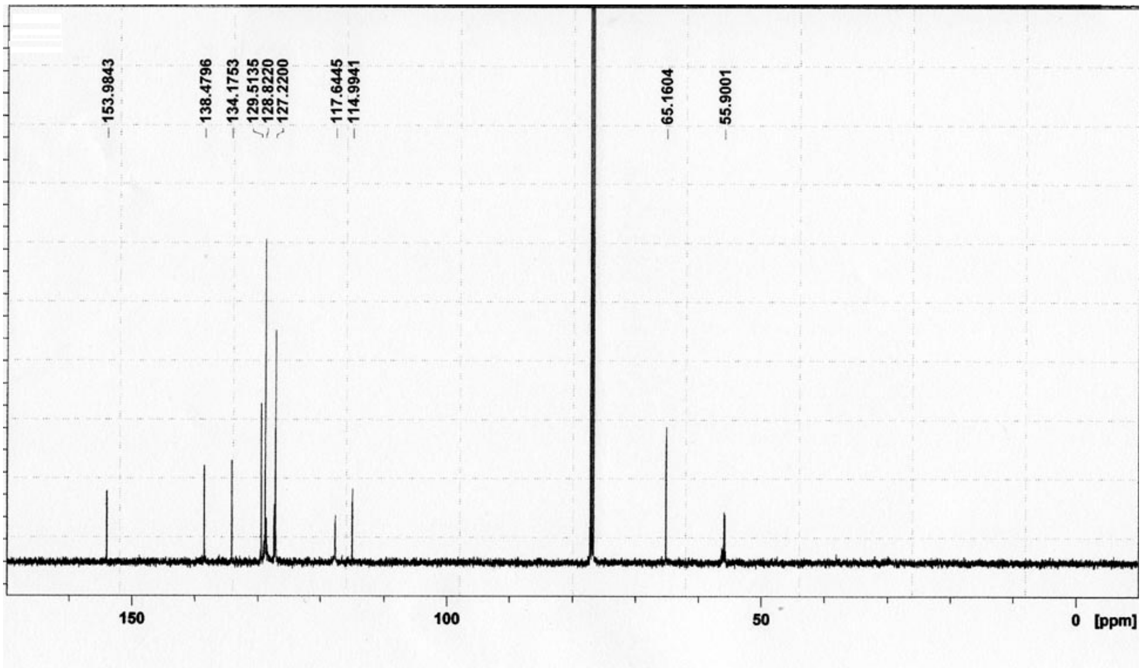

Figure S14. ^{13}C-NMR Spectrum of compound **2g**..

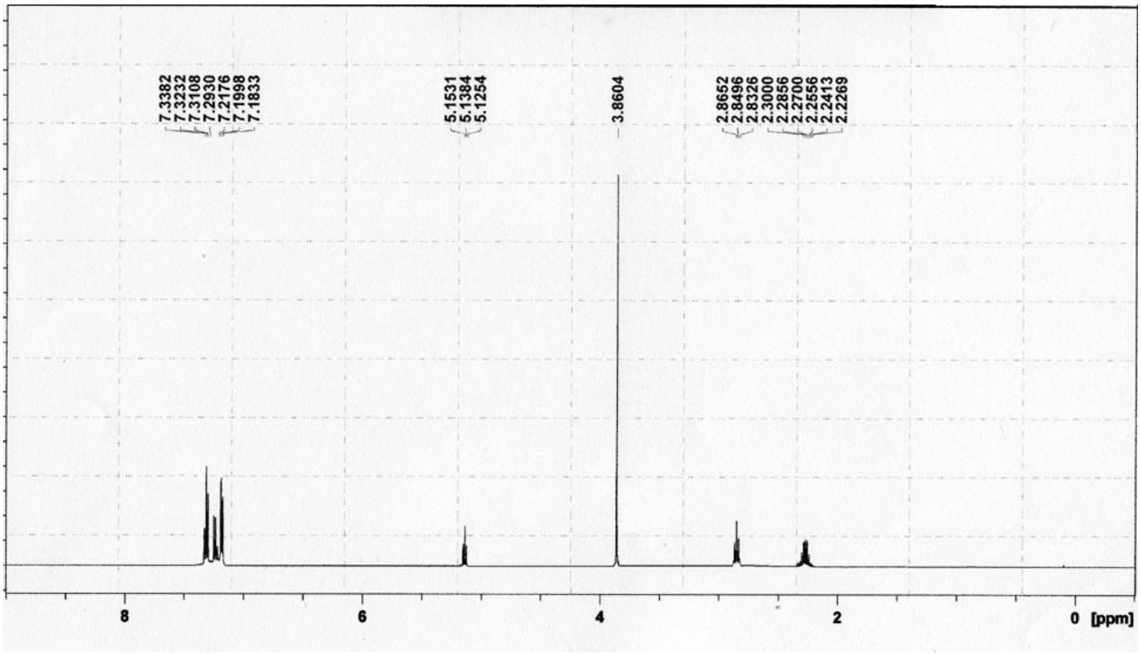

Figure S15. ^{1}H-NMR Spectrum of compound **2h**.

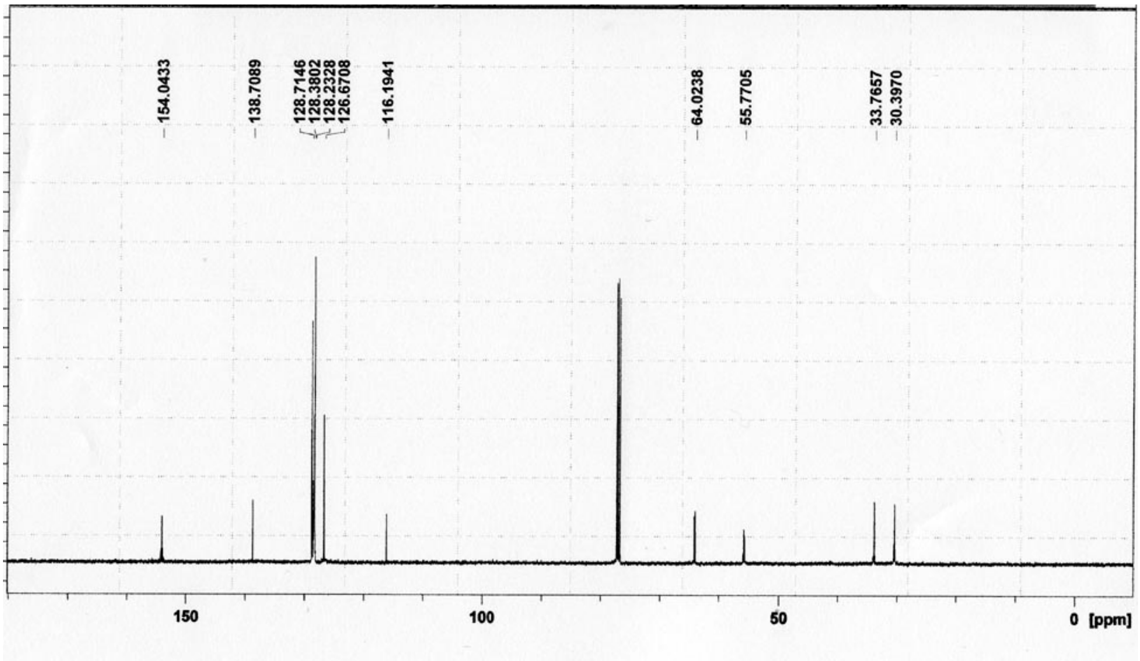

Figure S16. ^{13}C-NMR Spectrum of compound **2h**.

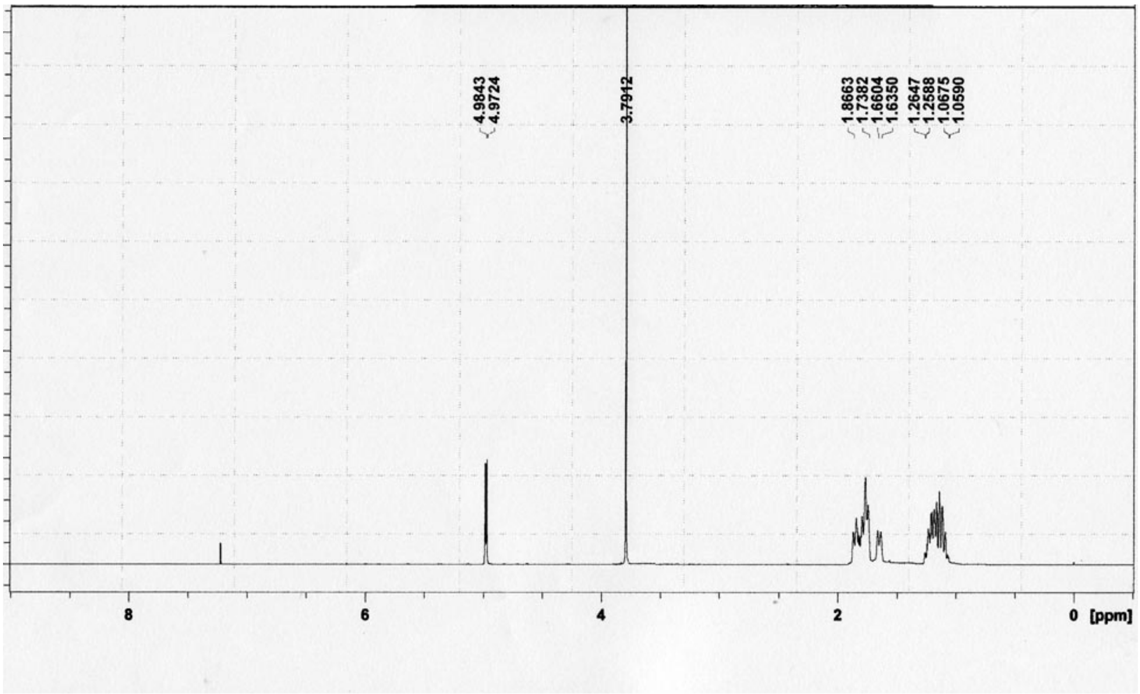

Figure S17. ^{1}H-NMR Spectrum of compound **2i**.

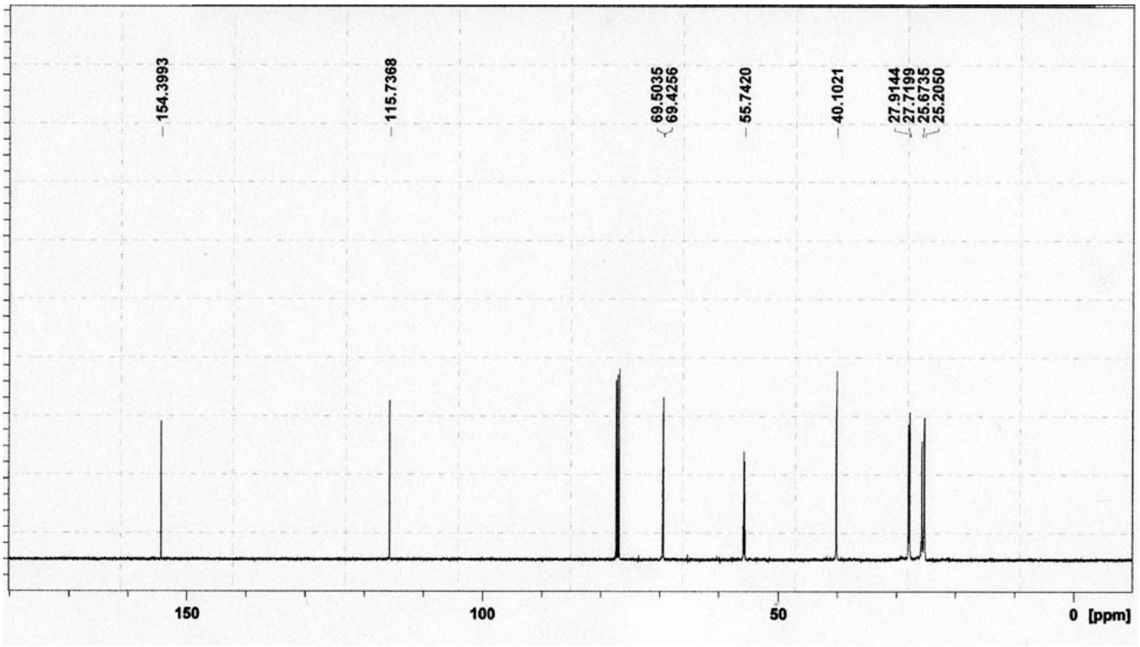

Figure S18. ^{13}C-NMR Spectrum of compound **2i**.

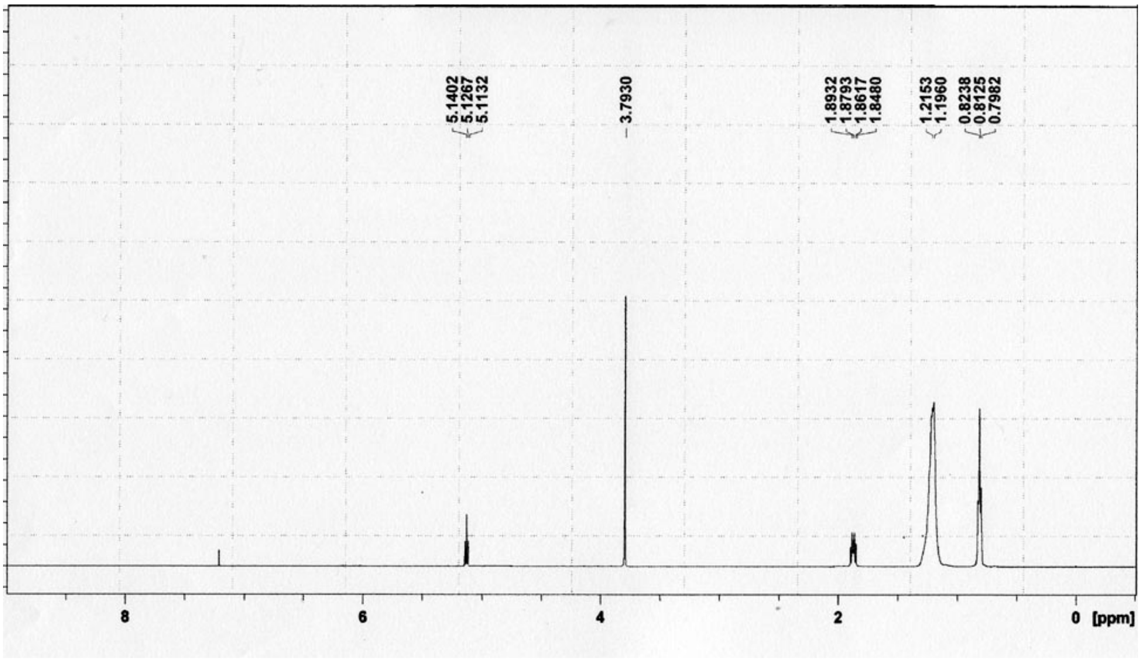

Figure S19. ^{1}H-NMR Spectrum of compound **2j**.

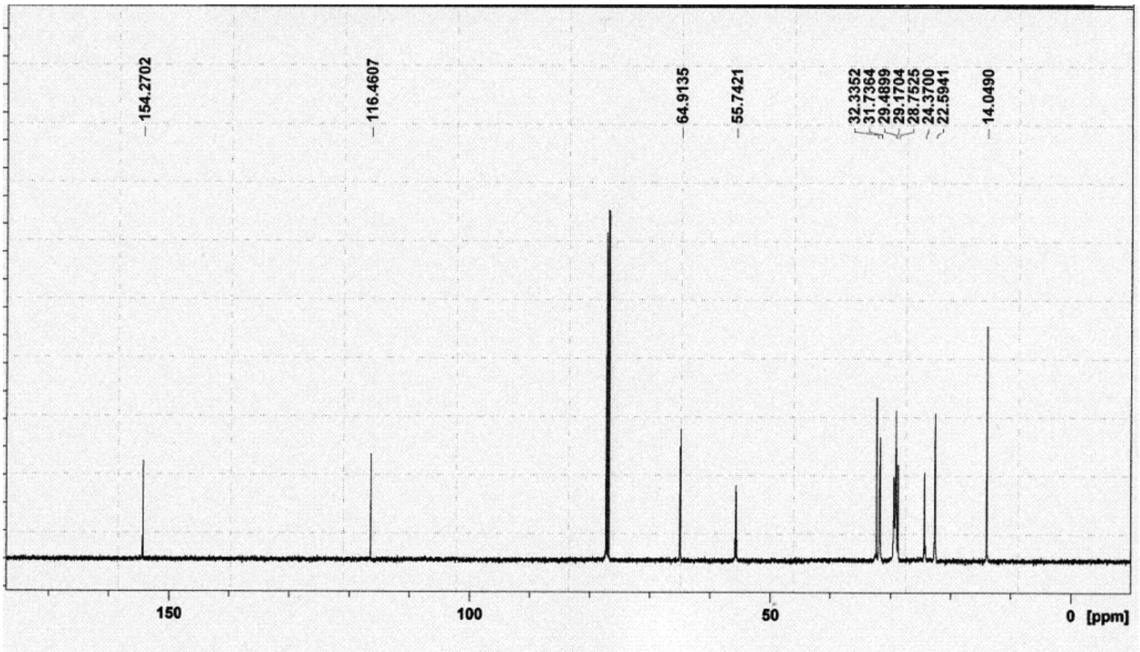

Figure S20. ^{13}C-NMR Spectrum of compound **2j**.

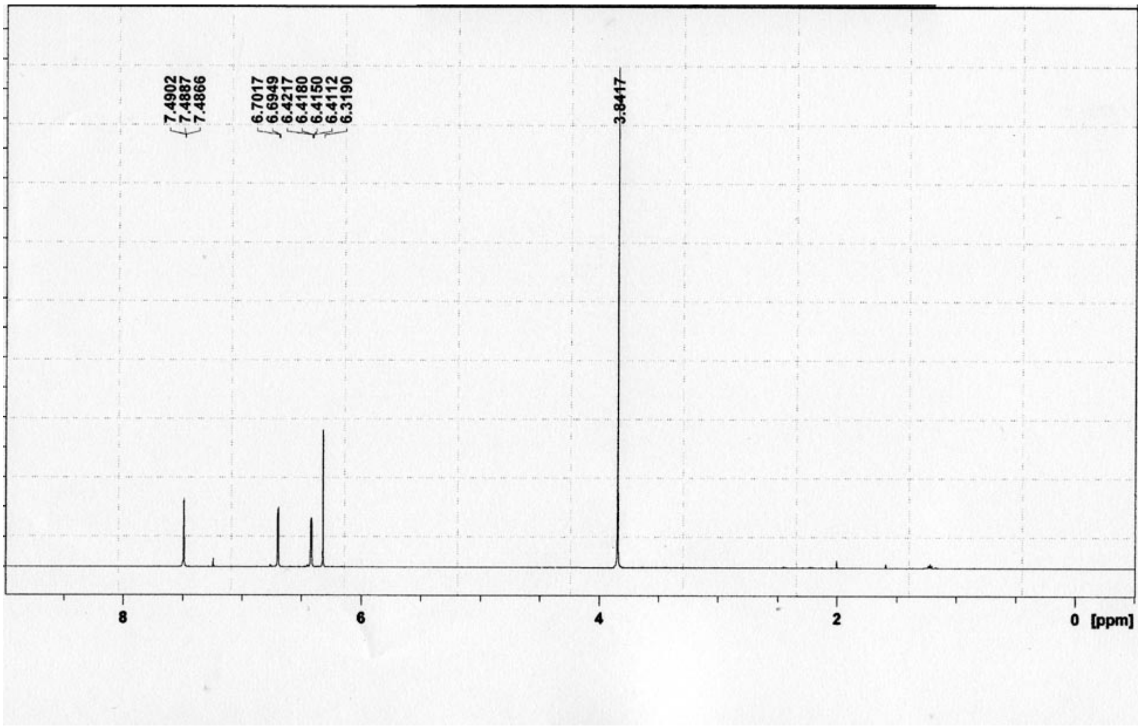

Figure S21. ^{1}H-NMR Spectrum of compound **2k**.

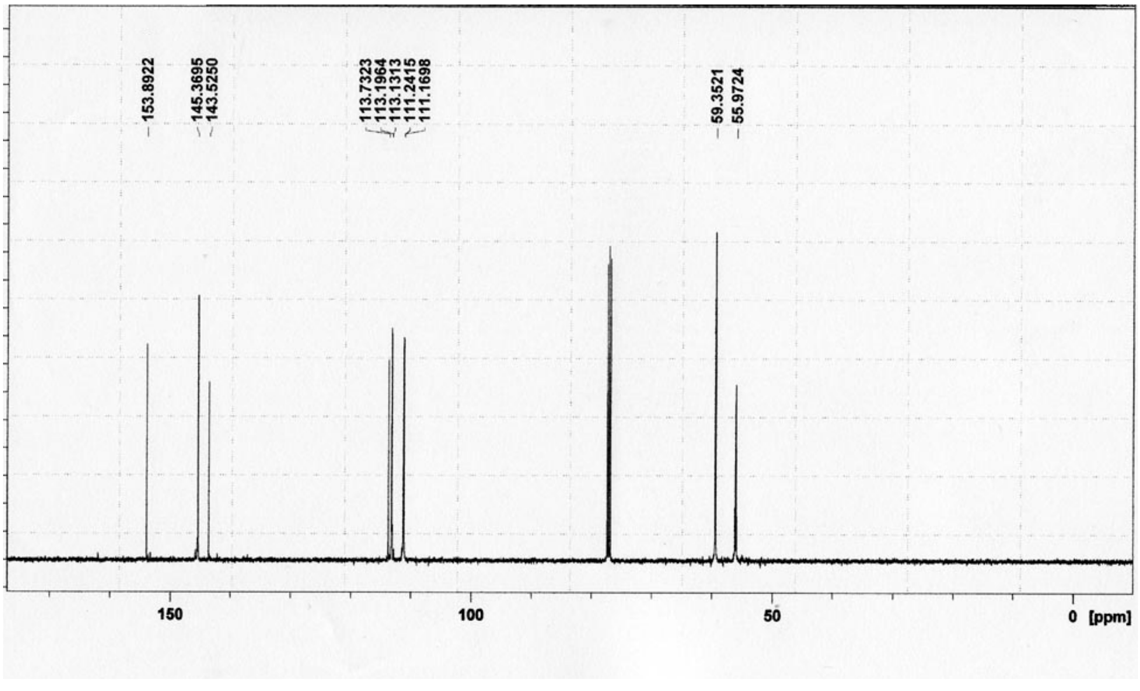

Figure S22. ^{13}C-NMR Spectrum of compound **2k**.

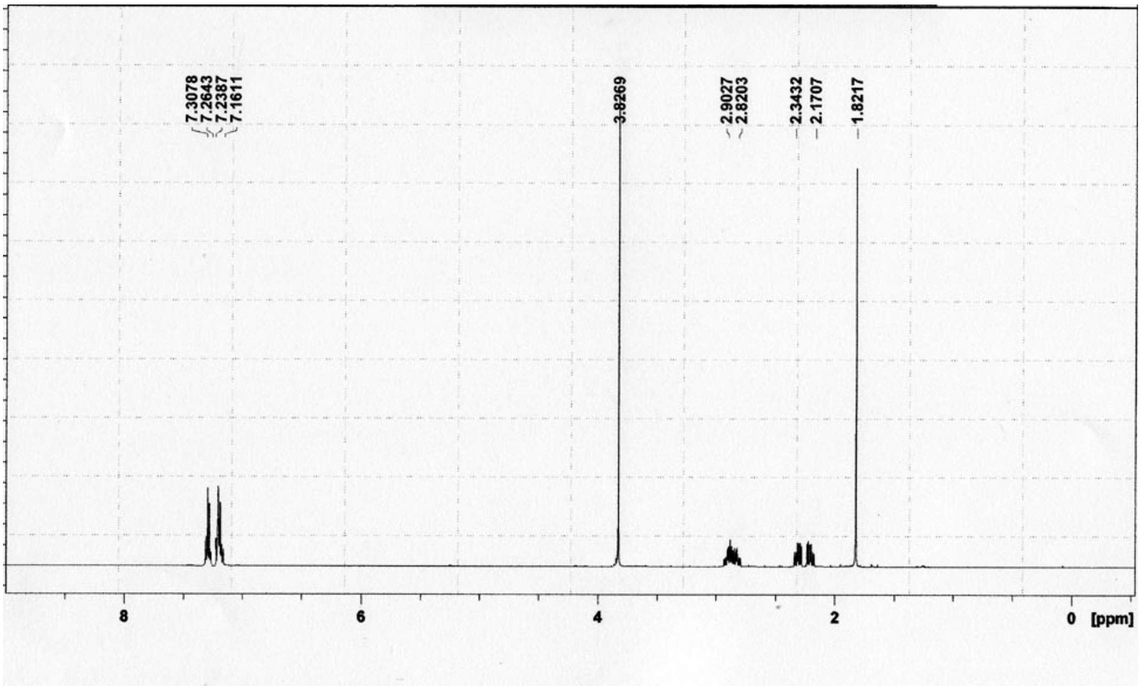

Figure S23. ^{1}H-NMR Spectrum of compound **4a**.

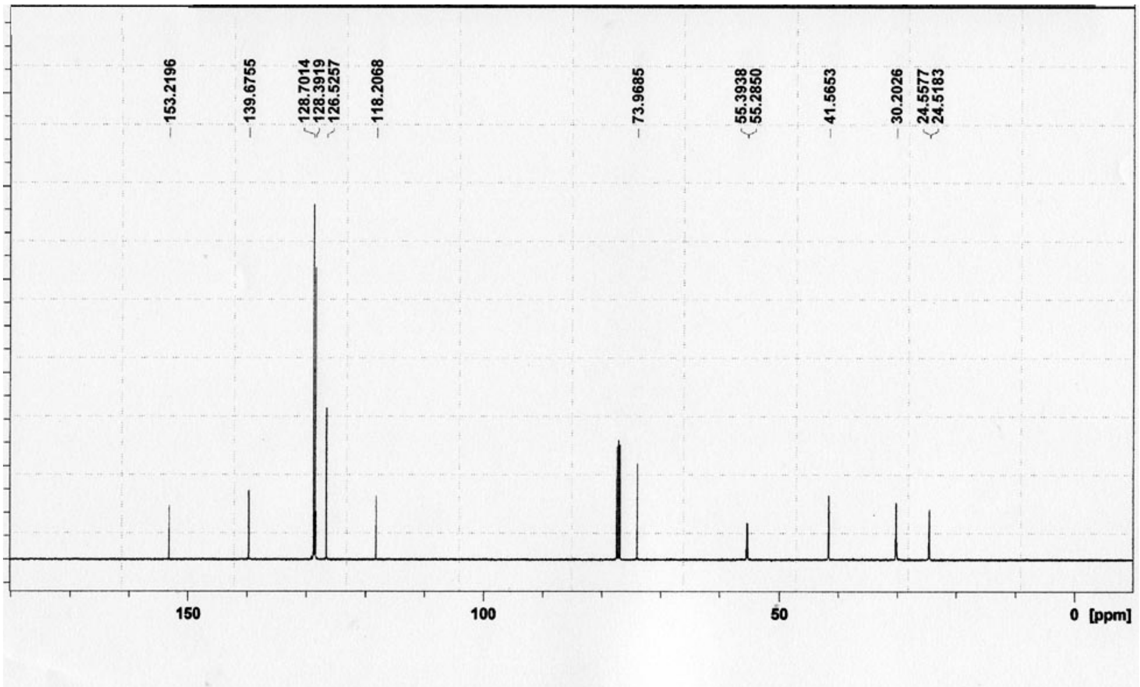

Figure S24. ^{13}C-NMR Spectrum of compound **4a**.

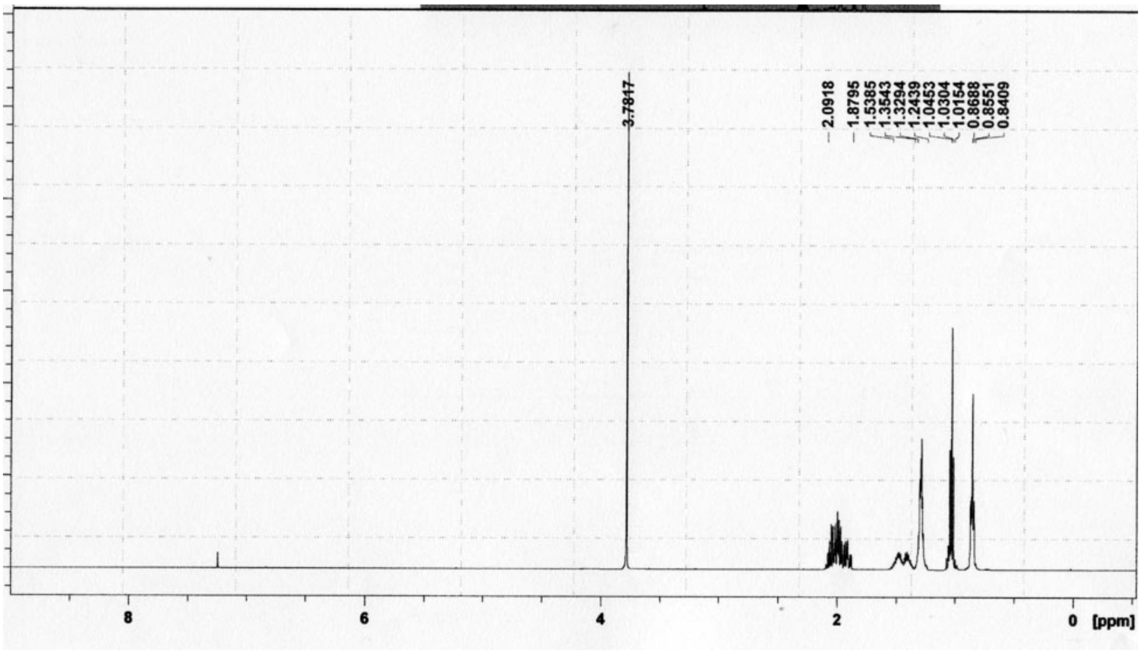

Figure S25. ^{1}H-NMR Spectrum of compound **4b**.

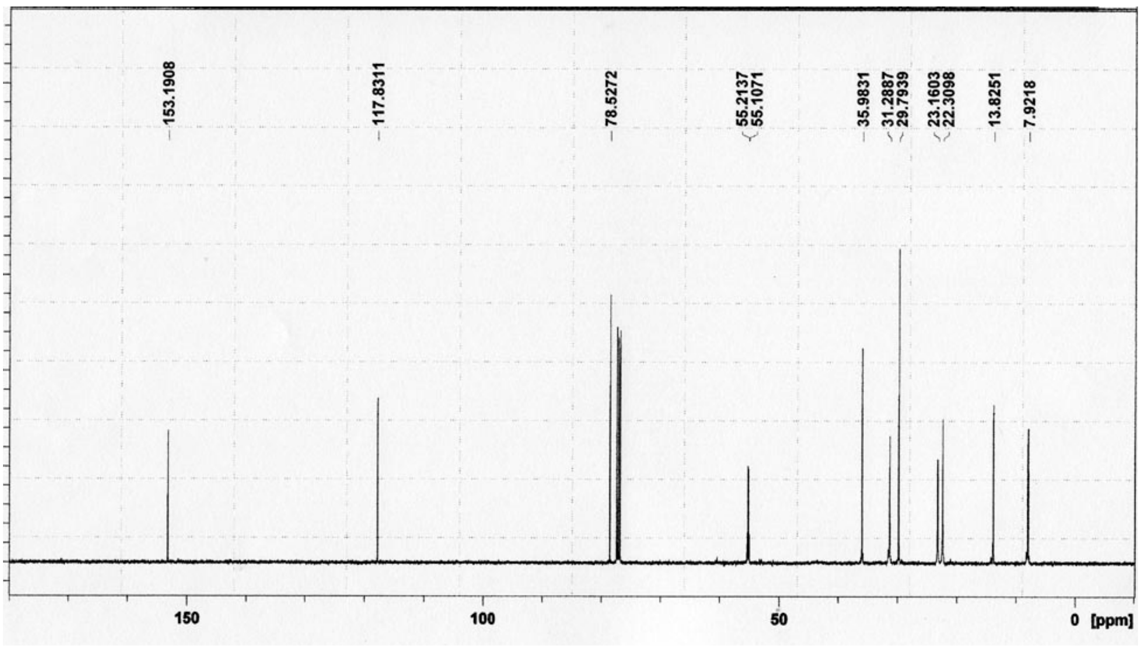

Figure S26. ^{13}C-NMR Spectrum of compound **4b**.

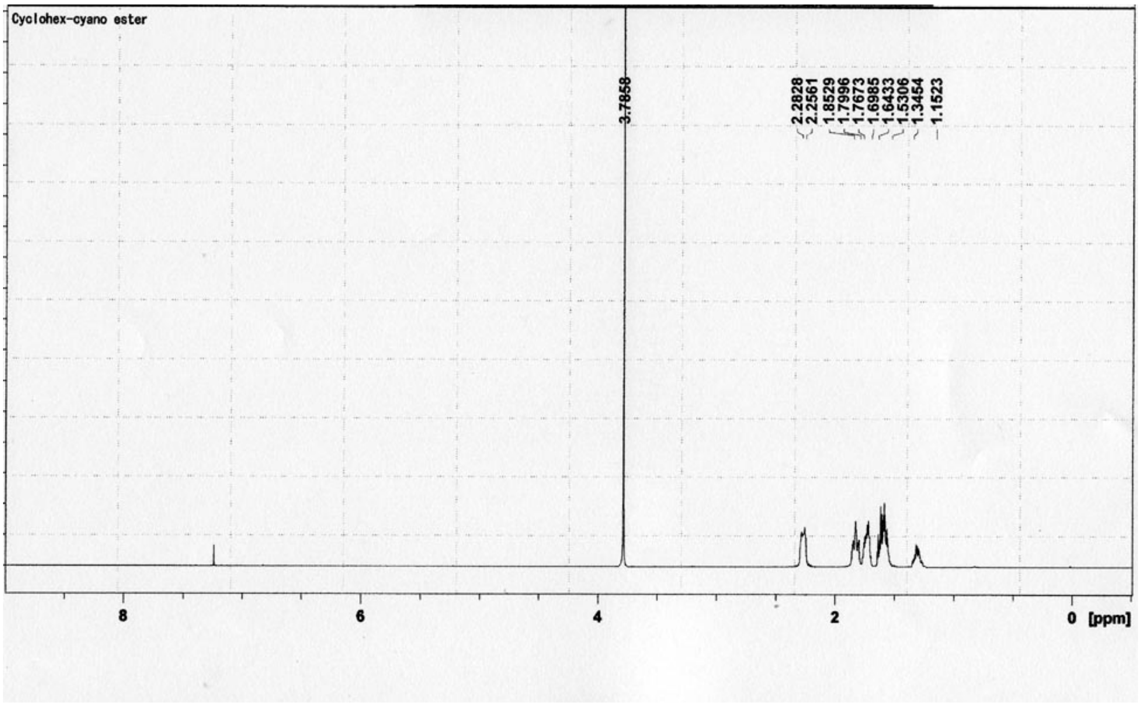

Figure S27. ¹H-NMR Spectrum of compound **4c**.

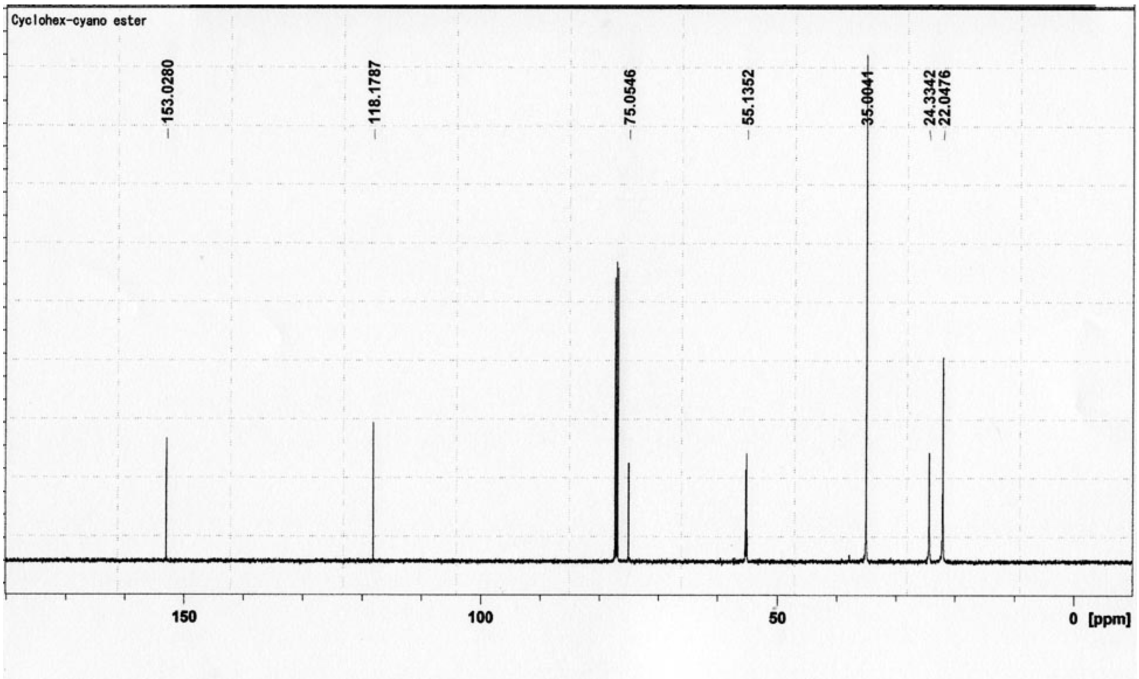

Figure S28. ¹³C-NMR Spectrum of compound **4c**.

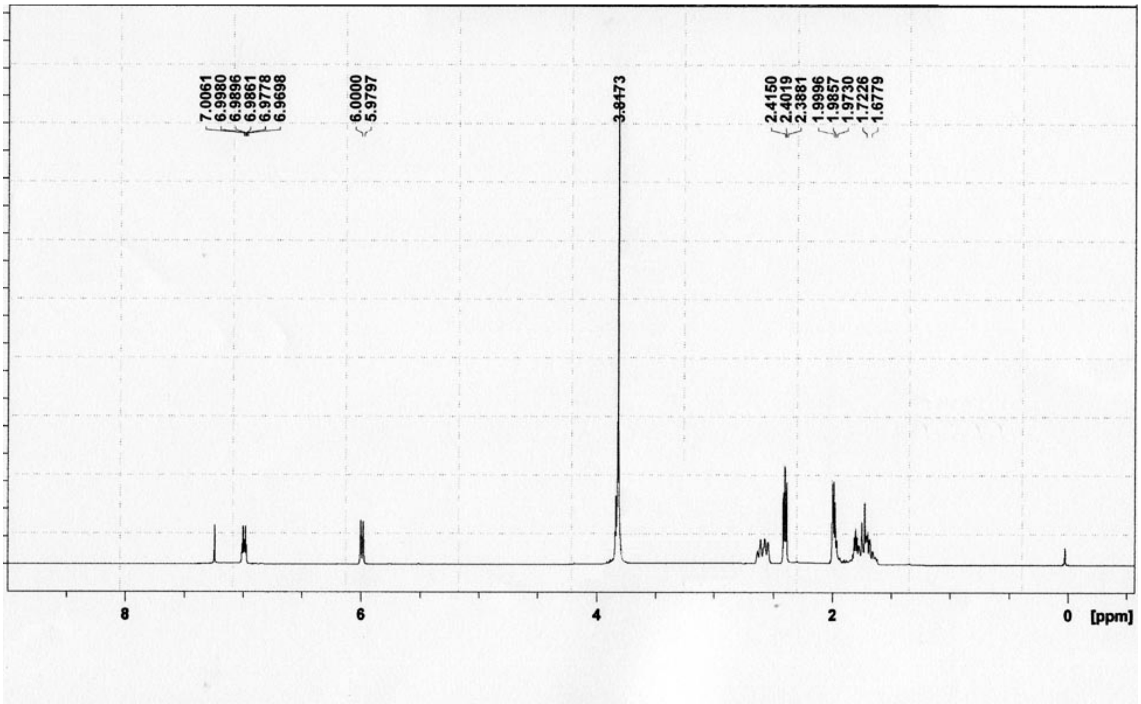

Figure S29. ^{1}H-NMR Spectrum of compound **4d**.

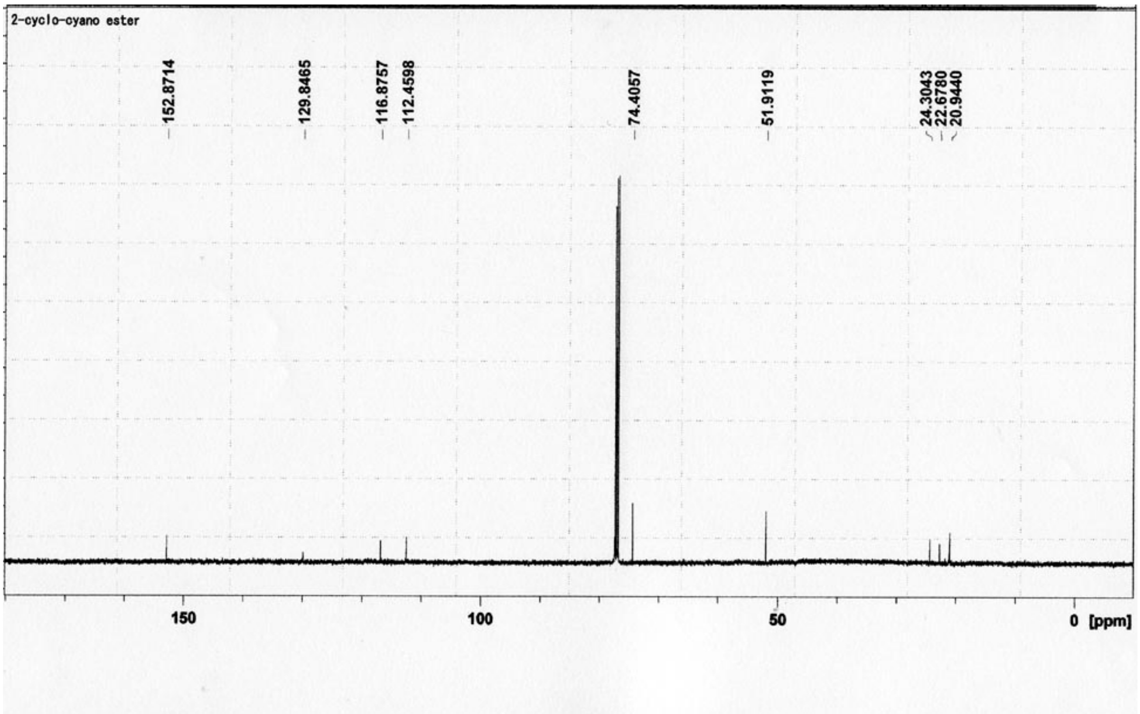

Figure S30. ^{13}C-NMR Spectrum of compound **4d**.

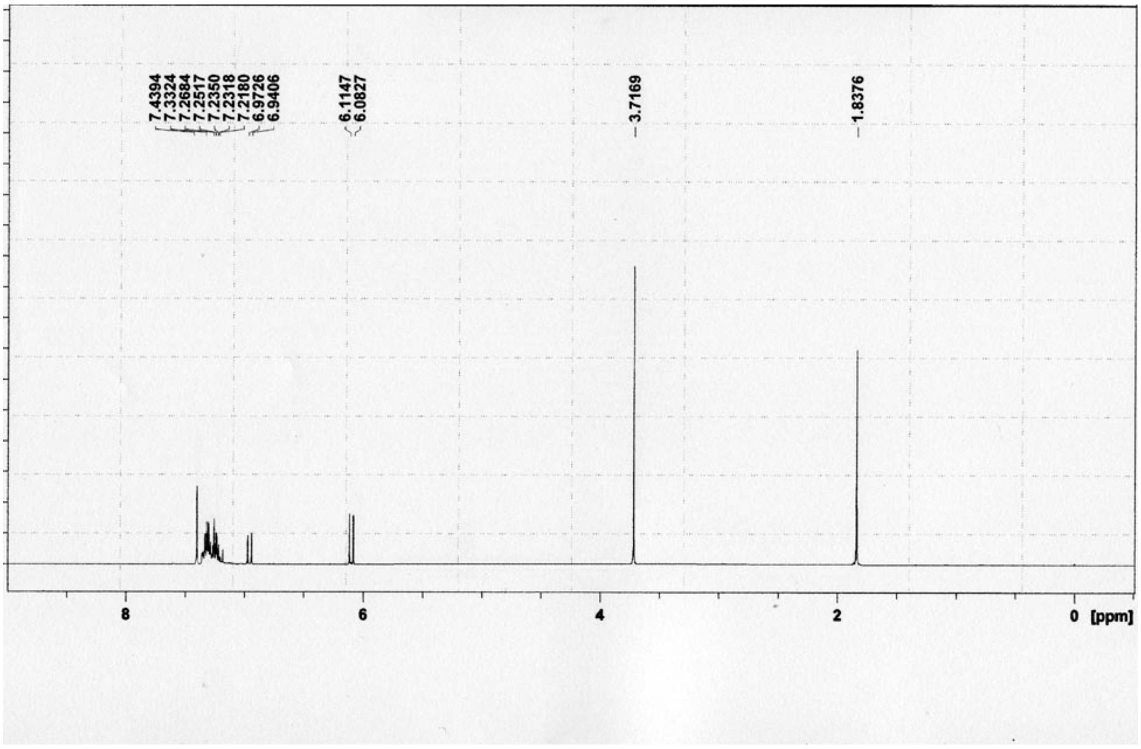

Figure S31. ^{1}H-NMR Spectrum of compound **4e**.

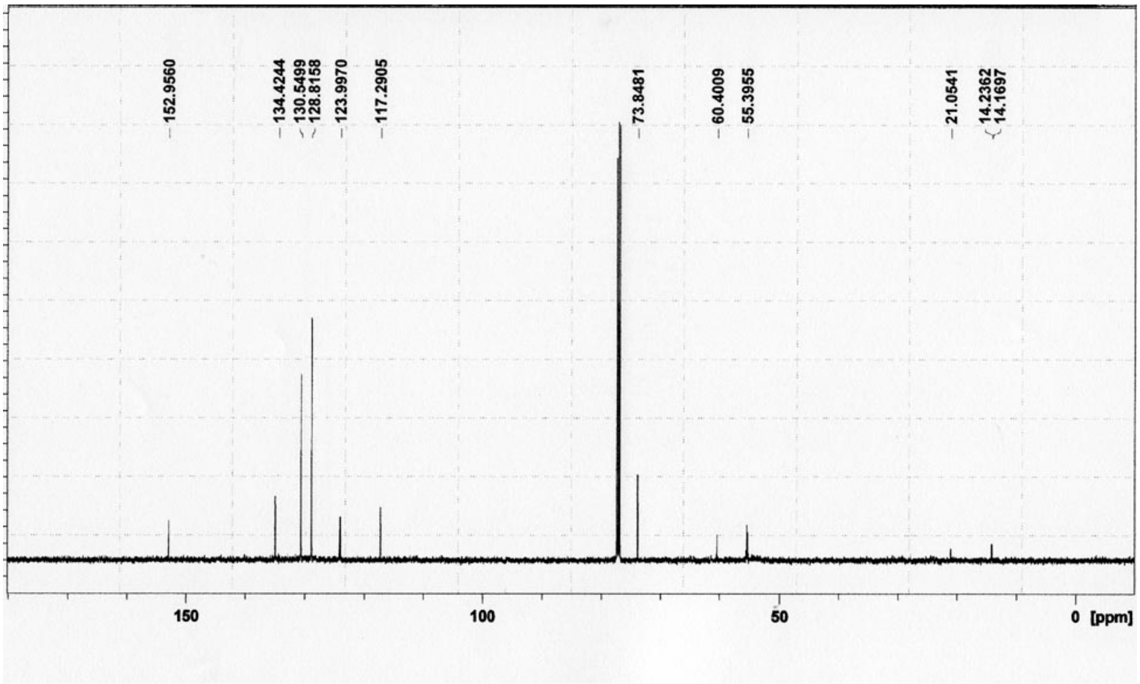

Figure S32. ^{13}C-NMR Spectrum of compound **4e**.

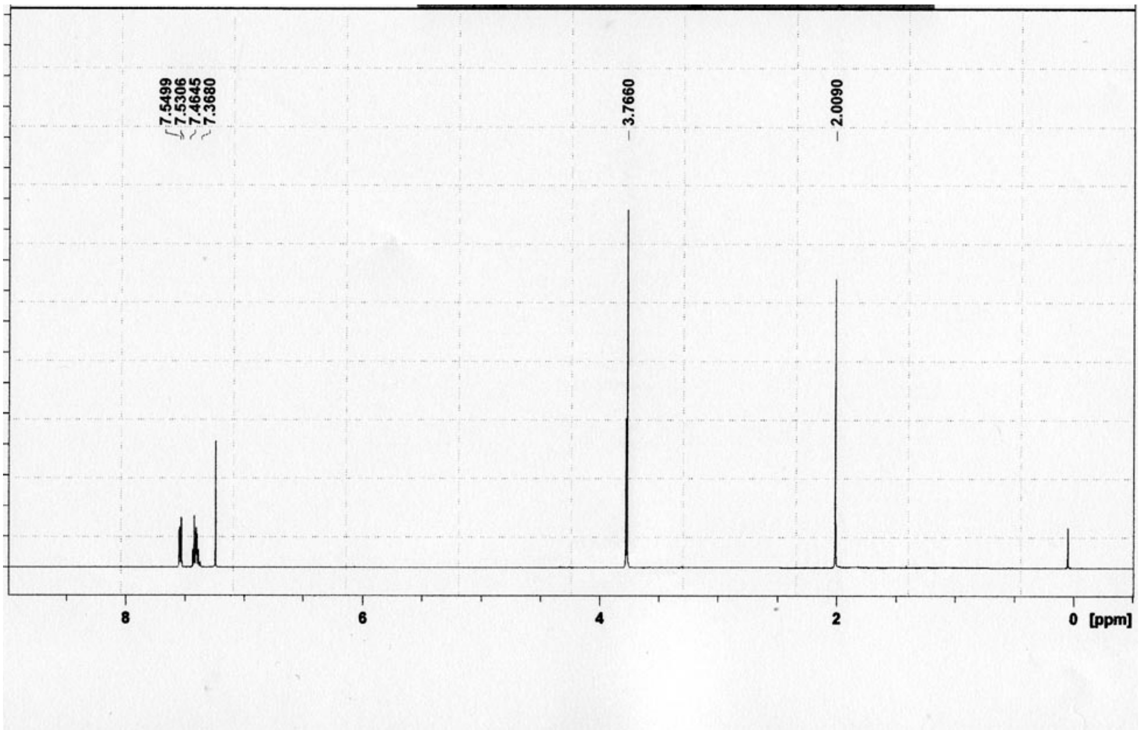

Figure S33. ^{1}H-NMR Spectrum of compound **4f**.

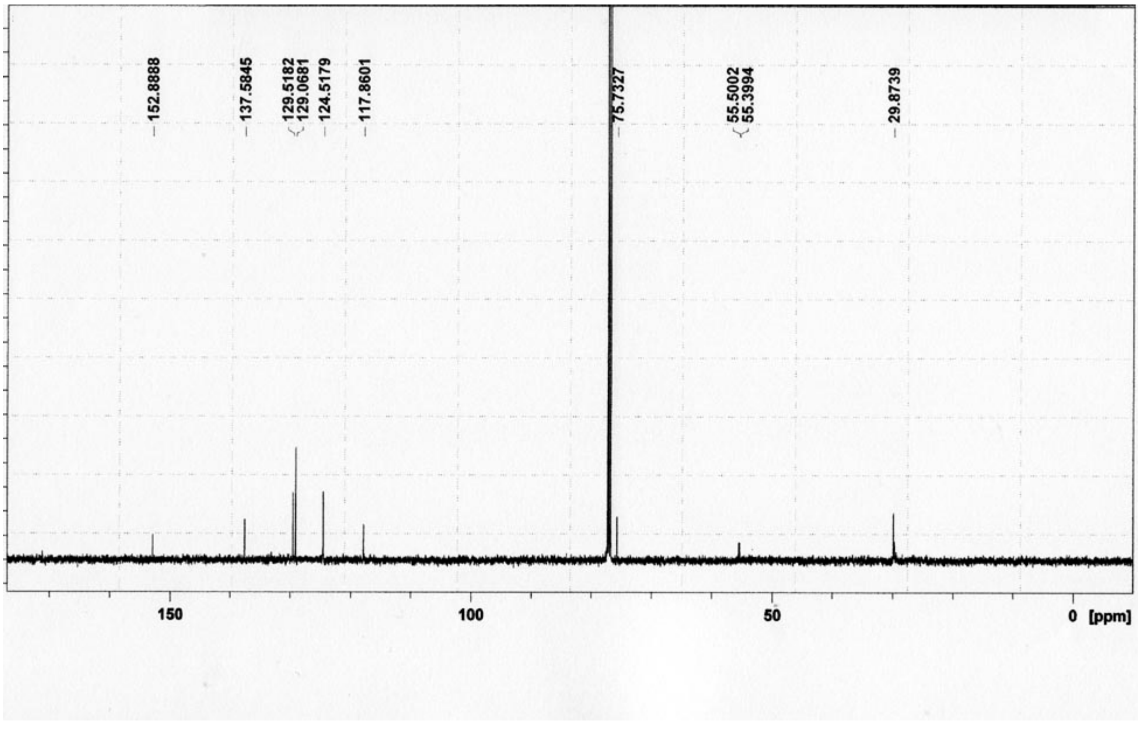

Figure S34. ^{13}C-NMR Spectrum of compound **4f**.

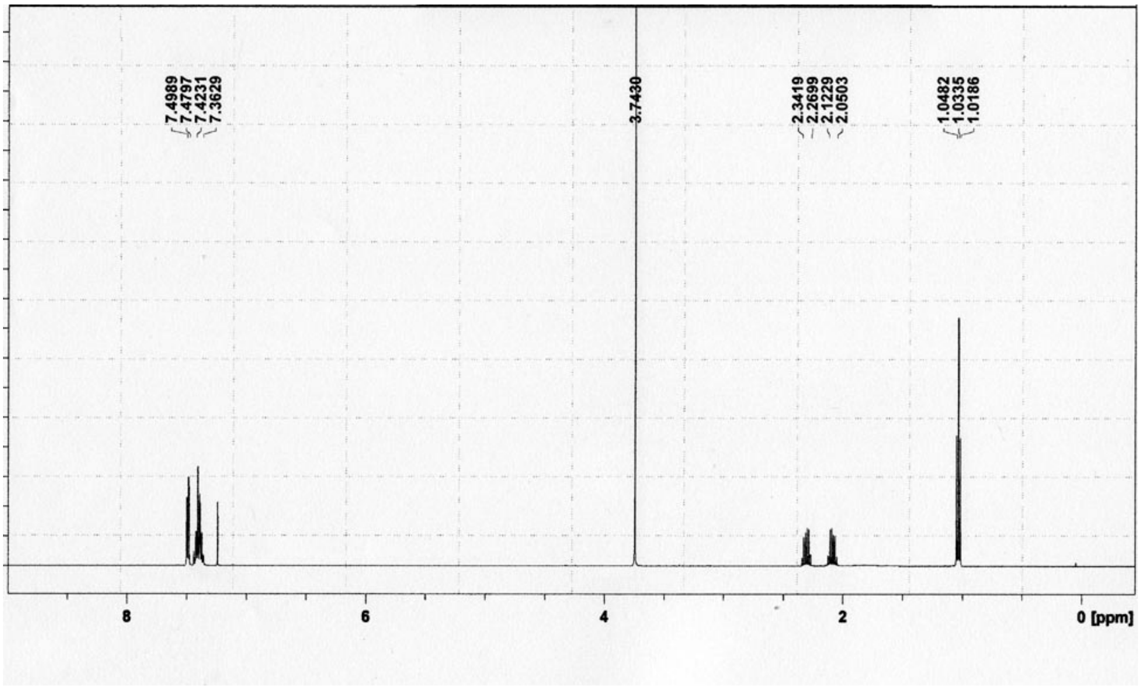

Figure S35. ^{1}H-NMR Spectrum of compound **4g**.

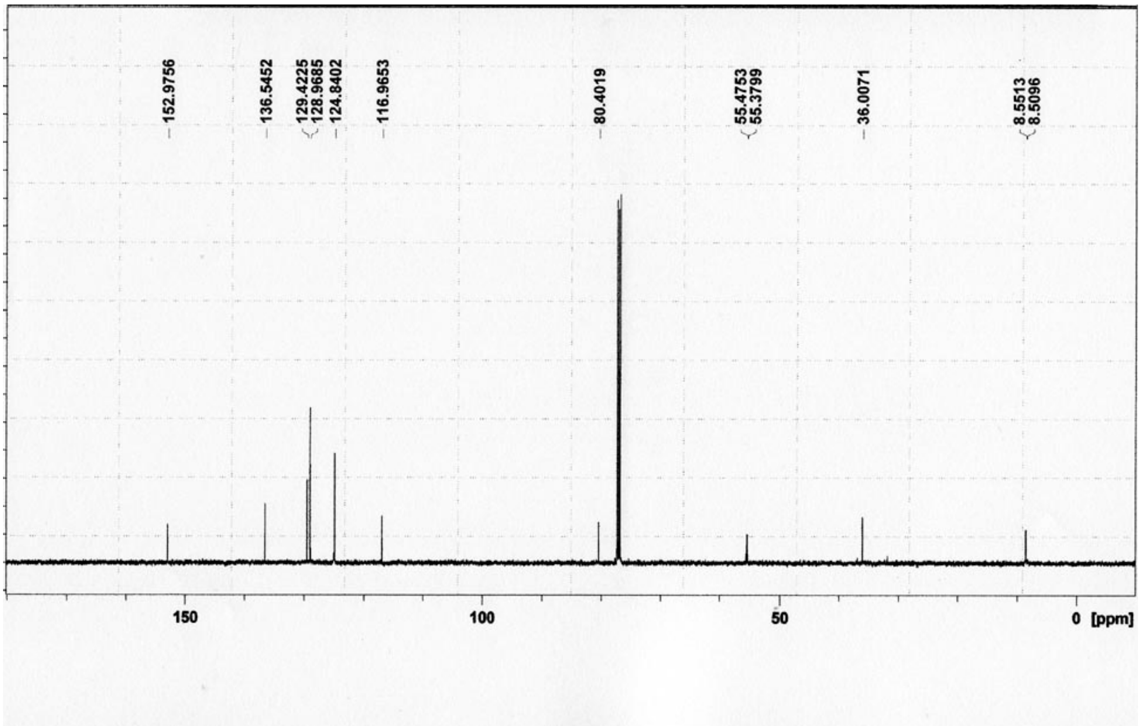

Figure S36. ^{13}C-NMR Spectrum of compound **4g**.

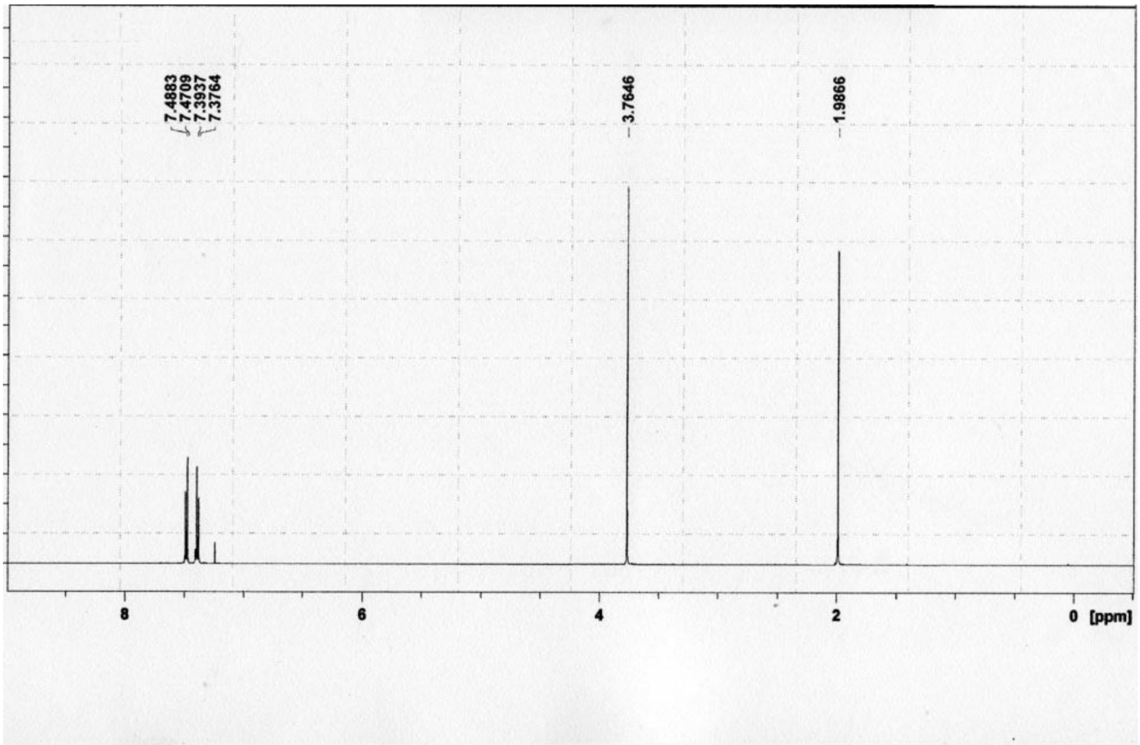

Figure S37. ^{1}H-NMR Spectrum of compound **4h**.

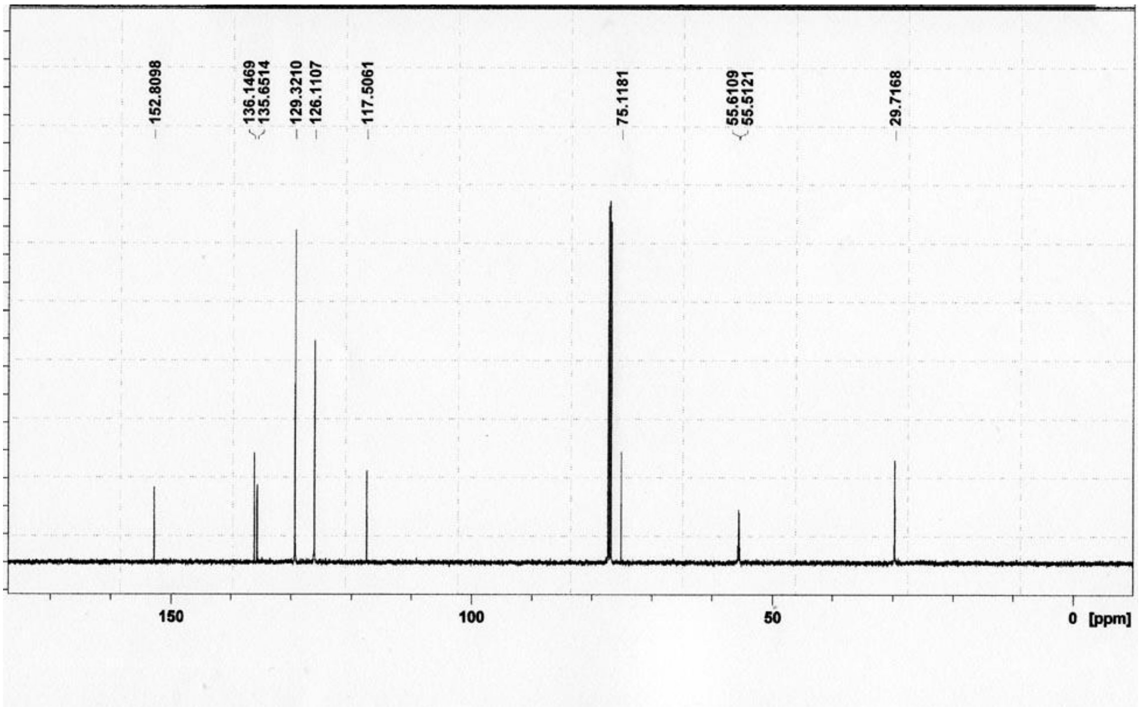

Figure S38. ^{13}C-NMR Spectrum of compound **4h**.

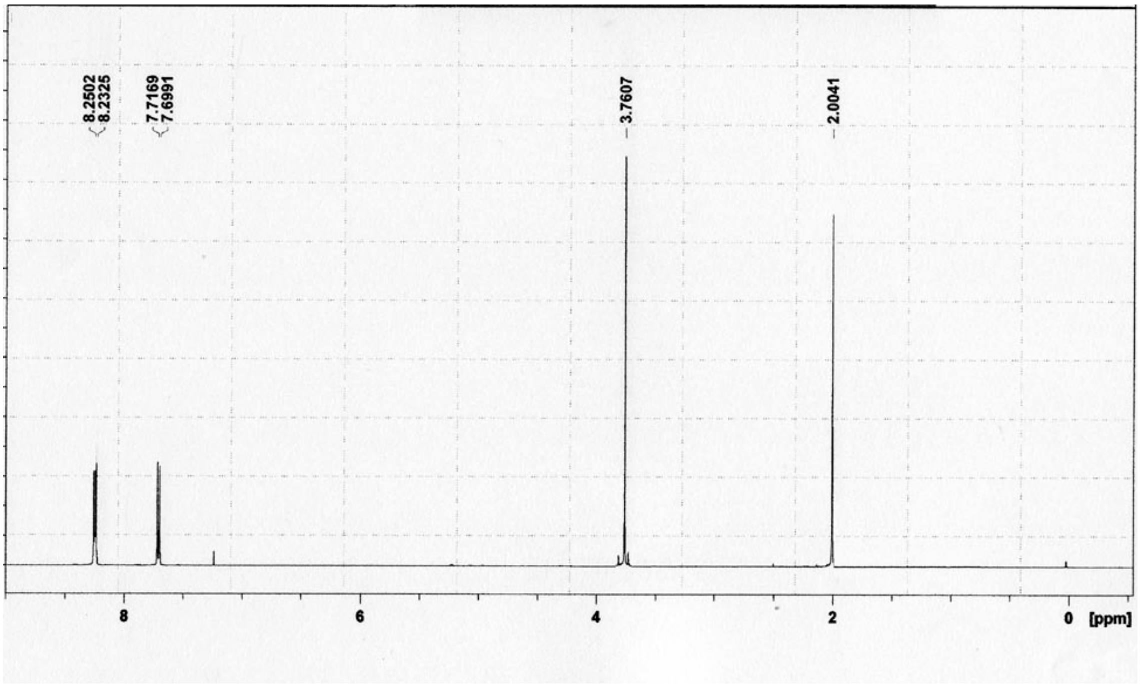

Figure S39. ^{1}H-NMR Spectrum of compound **4i**.

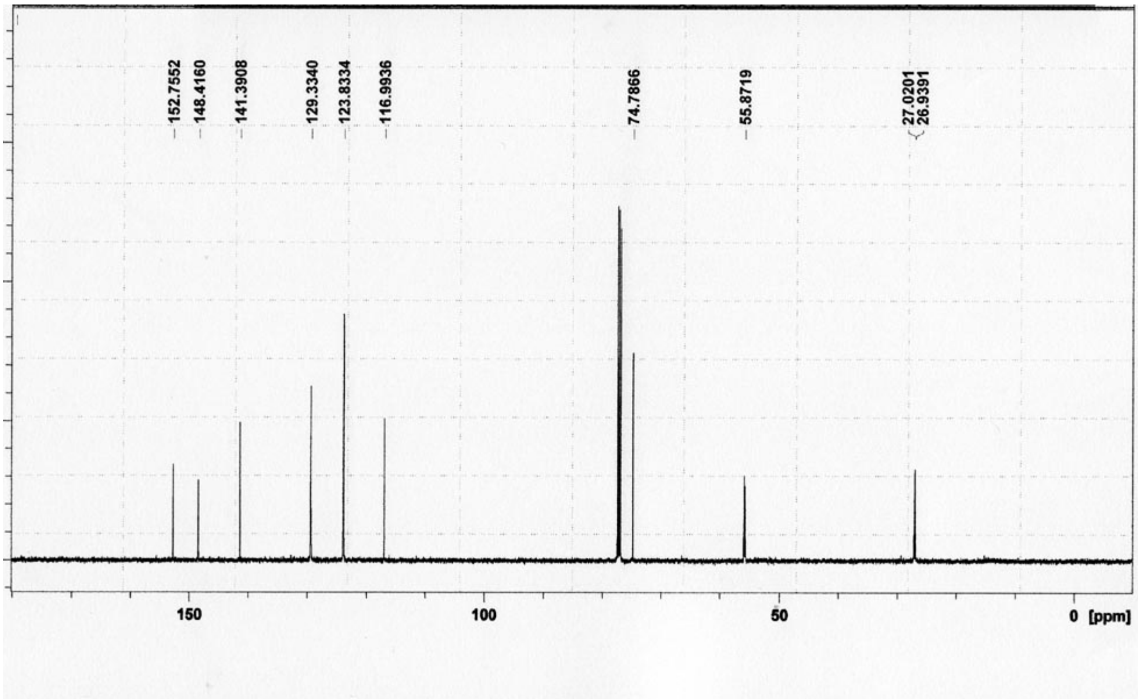

Figure S40. ^{13}C-NMR Spectrum of compound **4i**.

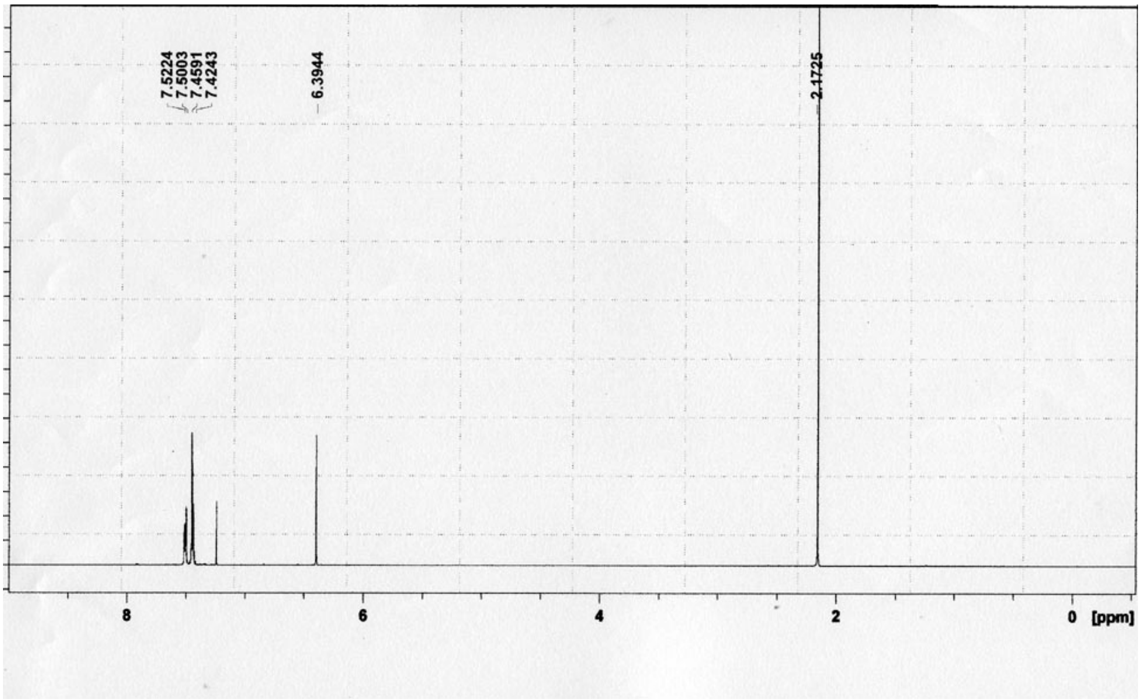

Figure S41. ^{1}H-NMR Spectrum of compound **5a**.

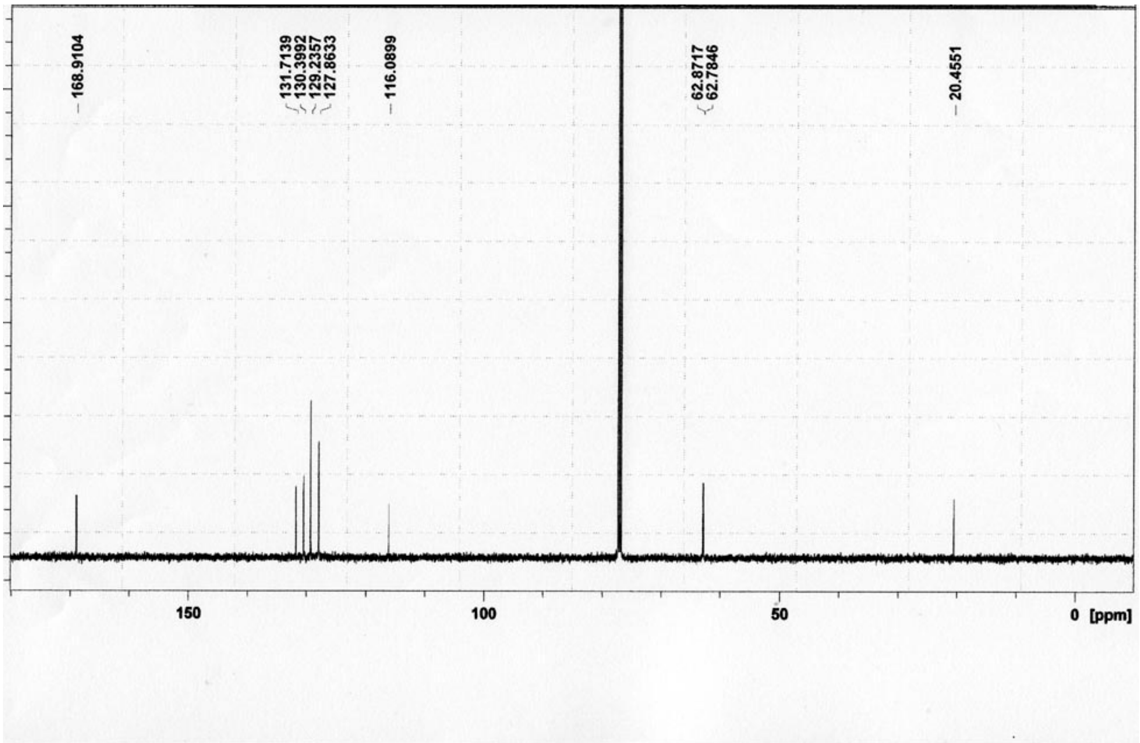

Figure S42. ^{13}C-NMR Spectrum of compound **5a**.

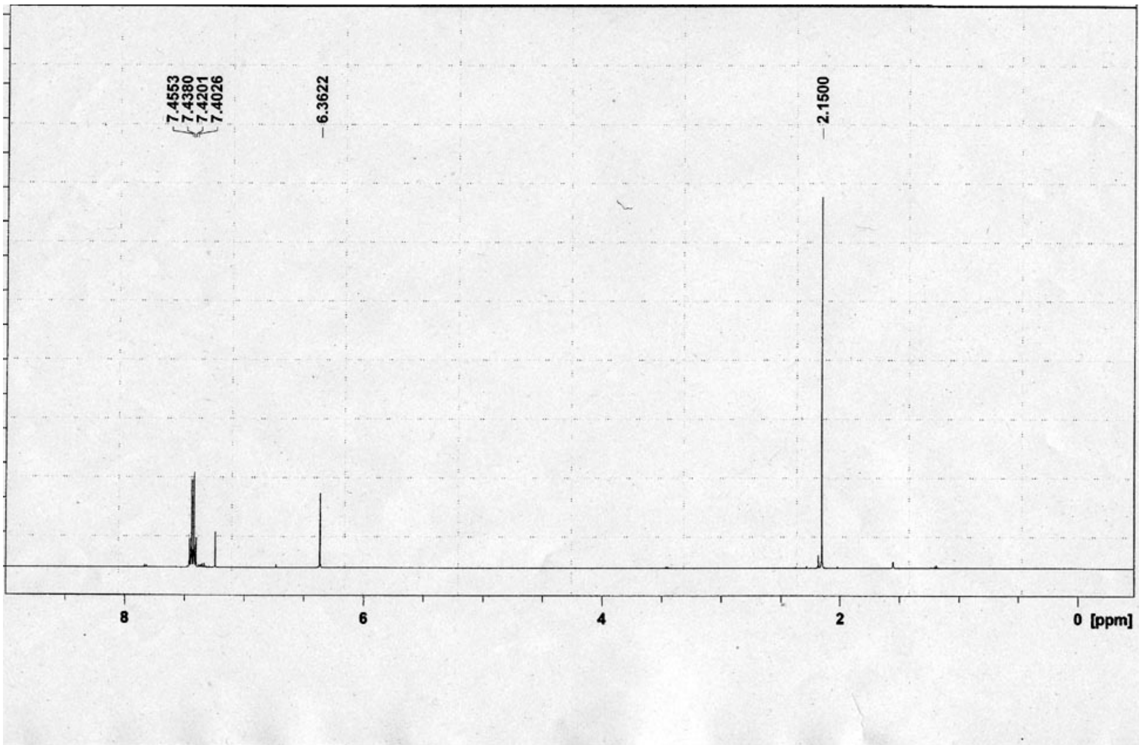

Figure S43. ^{1}H-NMR Spectrum of compound **5b**.

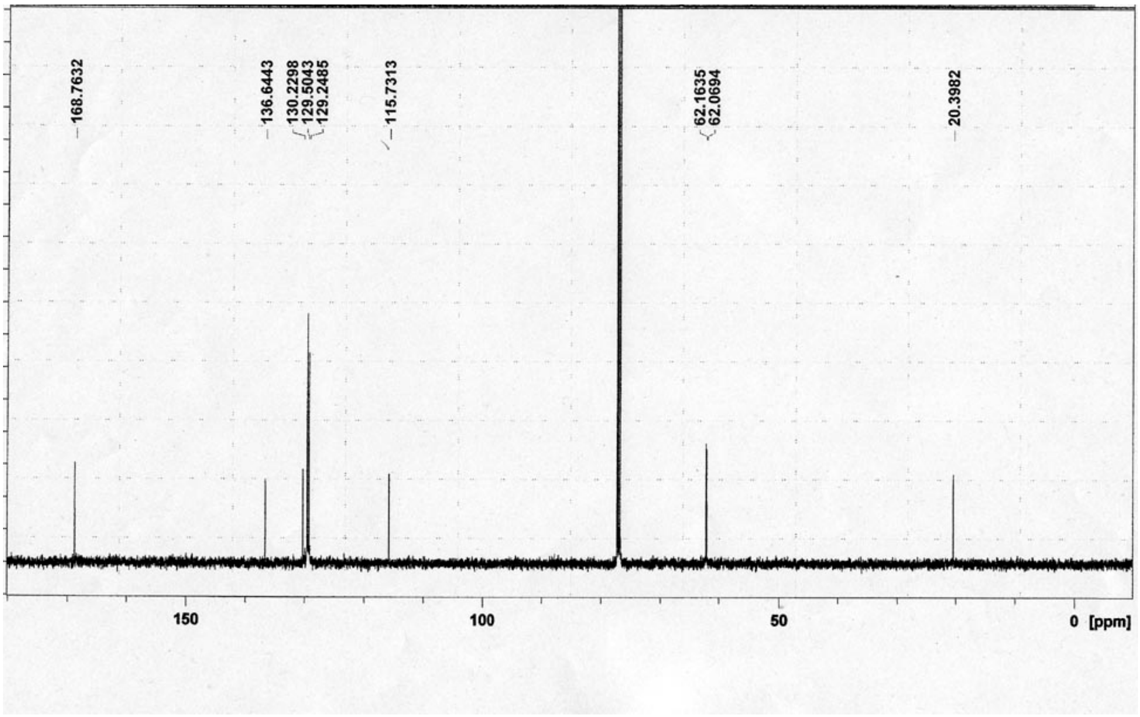

Figure S44. ^{13}C-NMR Spectrum of compound **5b**.

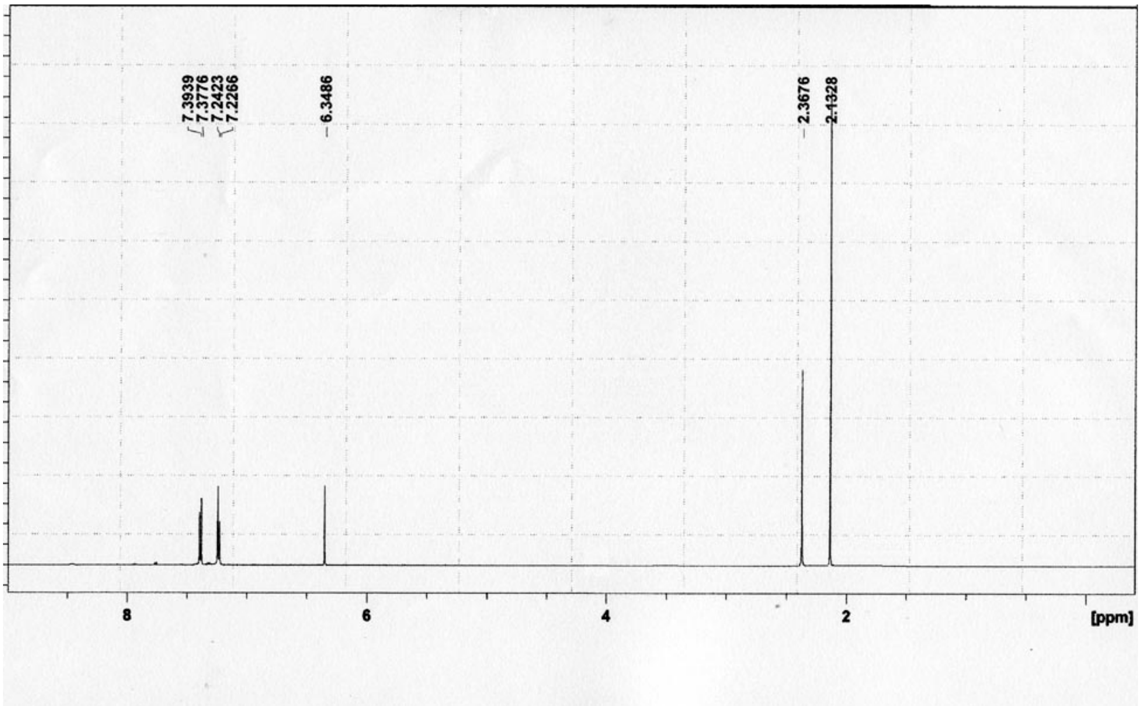

Figure S45. ^{1}H-NMR Spectrum of compound **5c**.

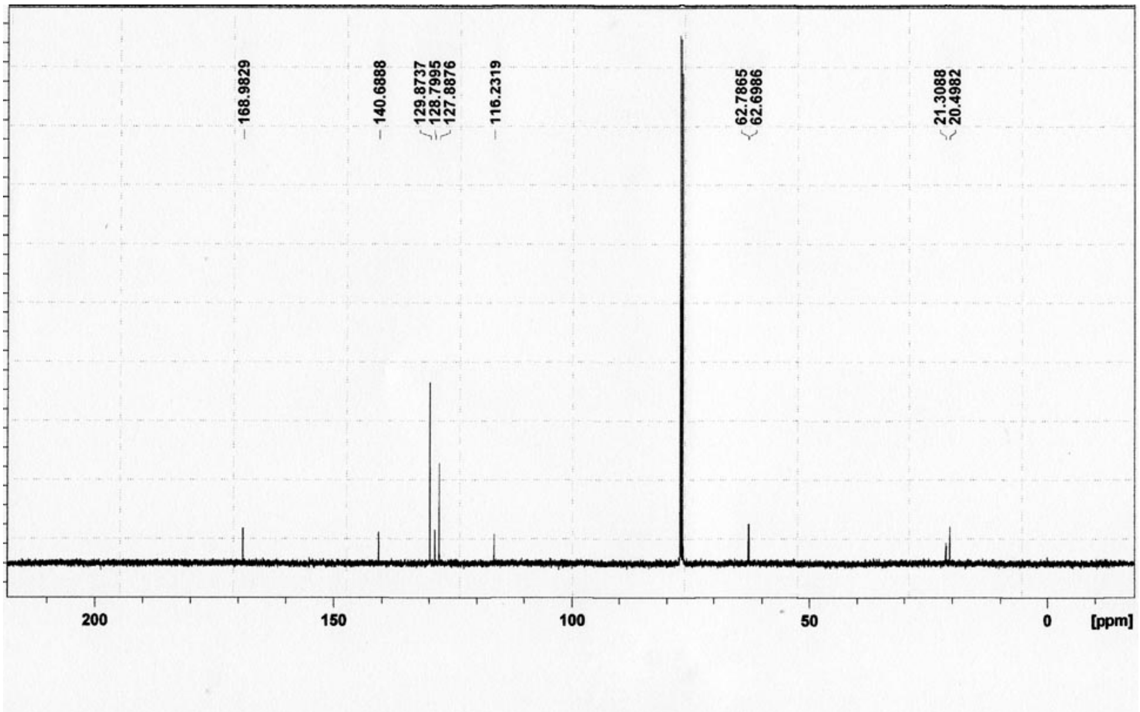

Figure S46. ^{13}C-NMR Spectrum of compound **5c**.

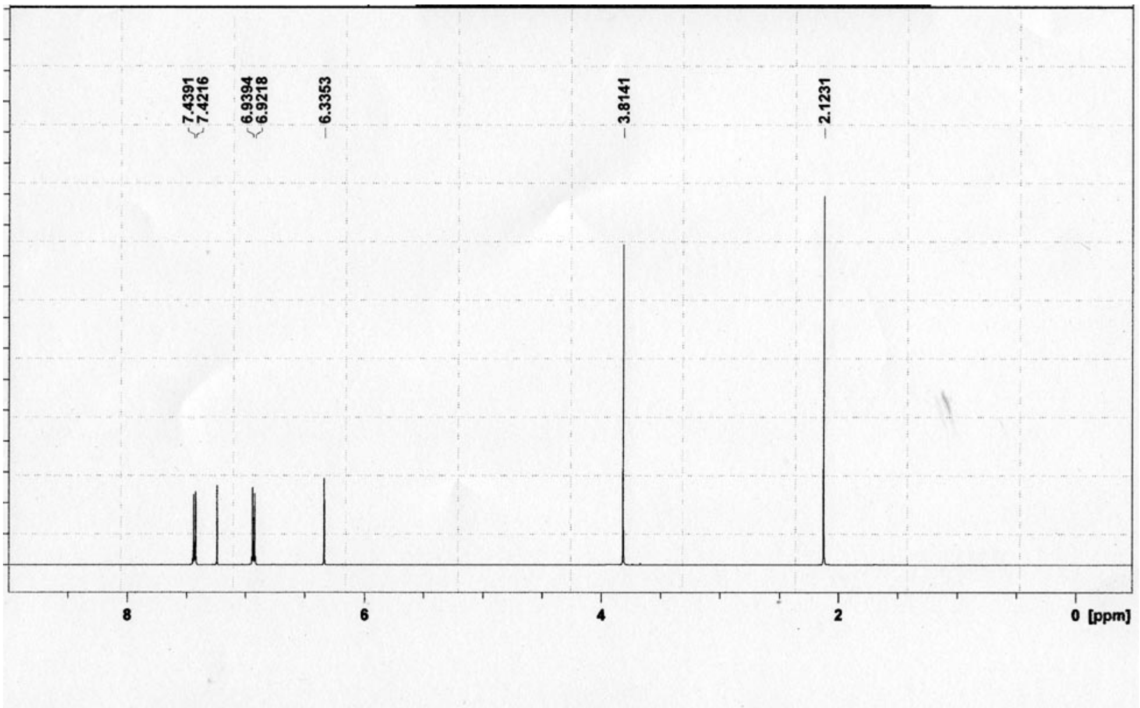

Figure S47. ^{1}H-NMR Spectrum of compound **5d**.

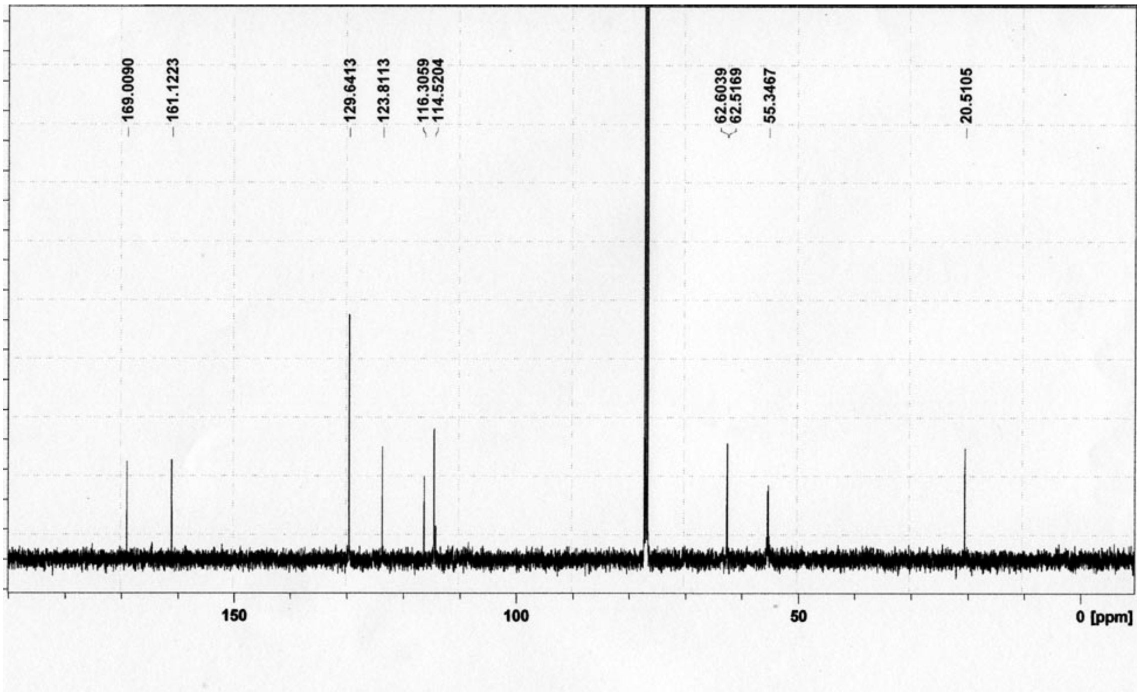

Figure S48. ^{13}C-NMR Spectrum of compound **5d**.

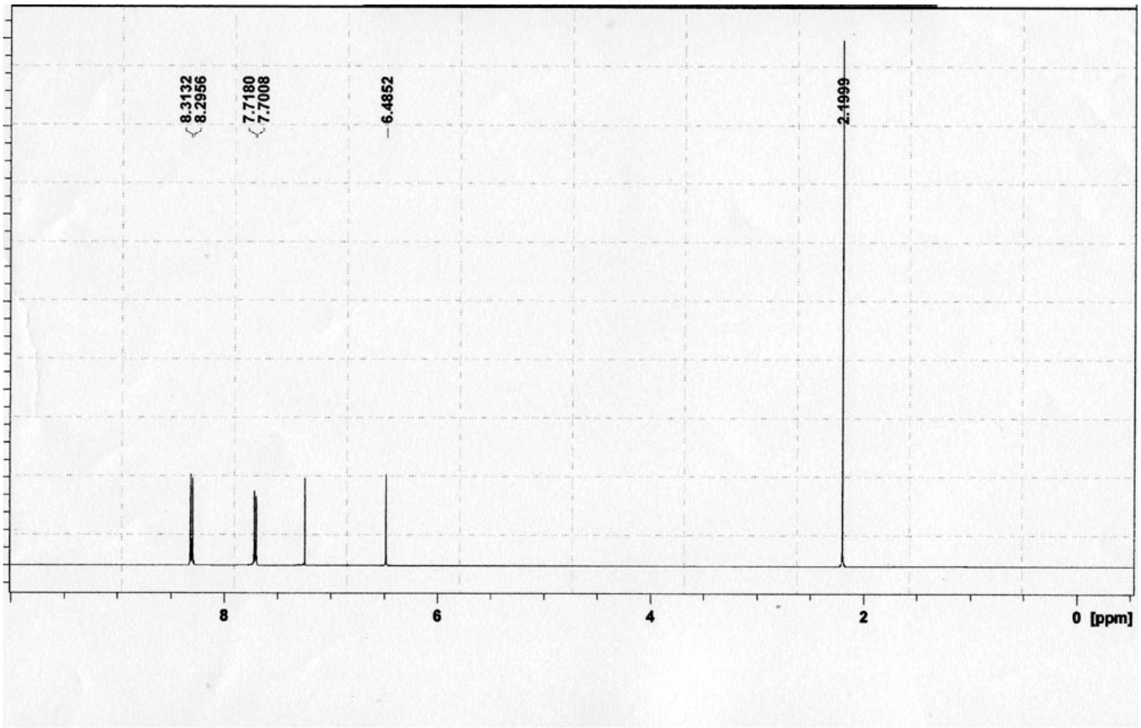

Figure S49. ^{1}H-NMR Spectrum of compound **5e**.

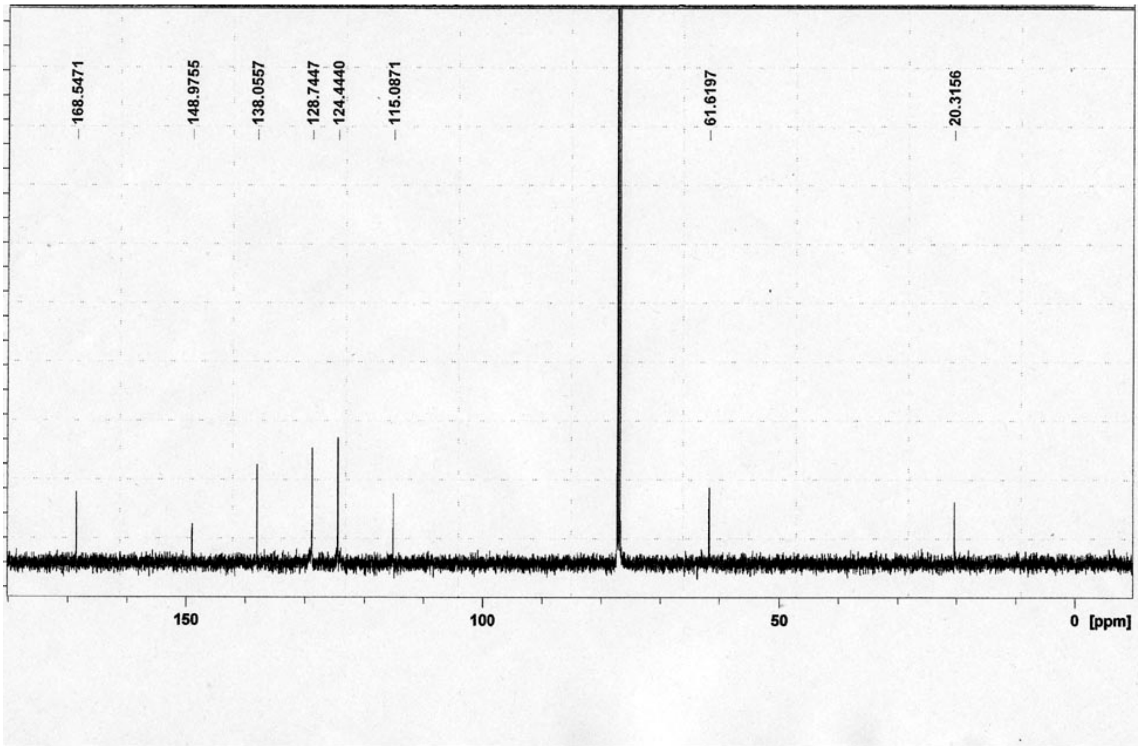

Figure S50. ^{13}C-NMR Spectrum of compound **5e**.

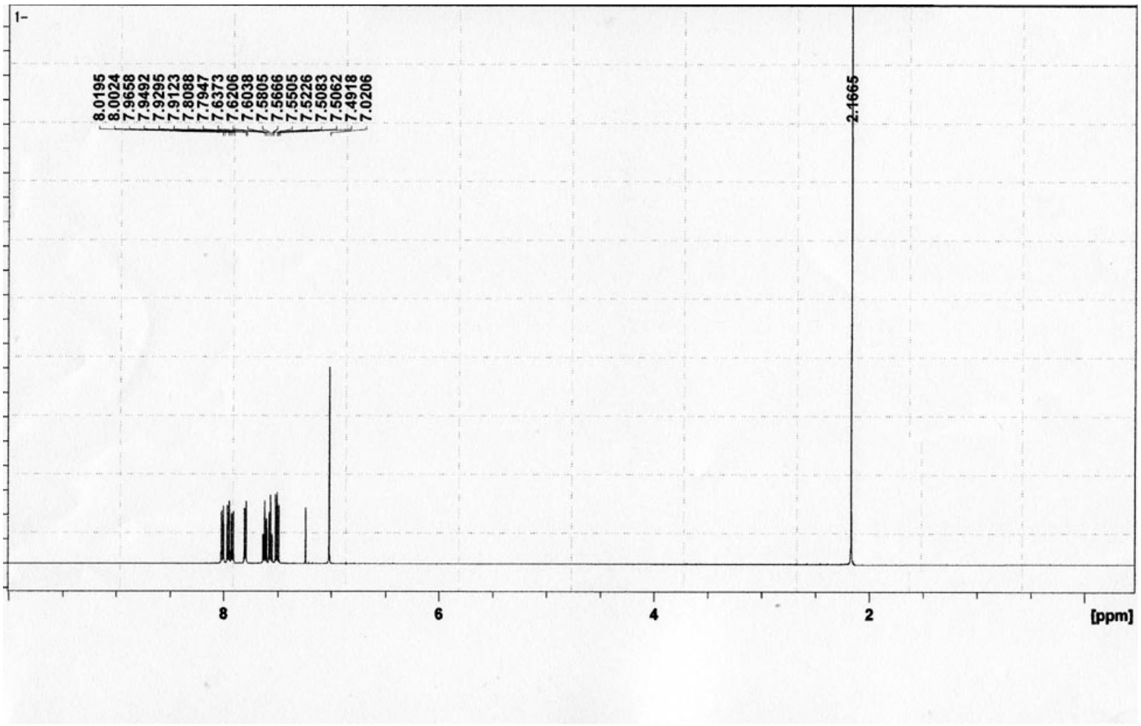

Figure S51. ^{1}H-NMR Spectrum of compound **5f**.

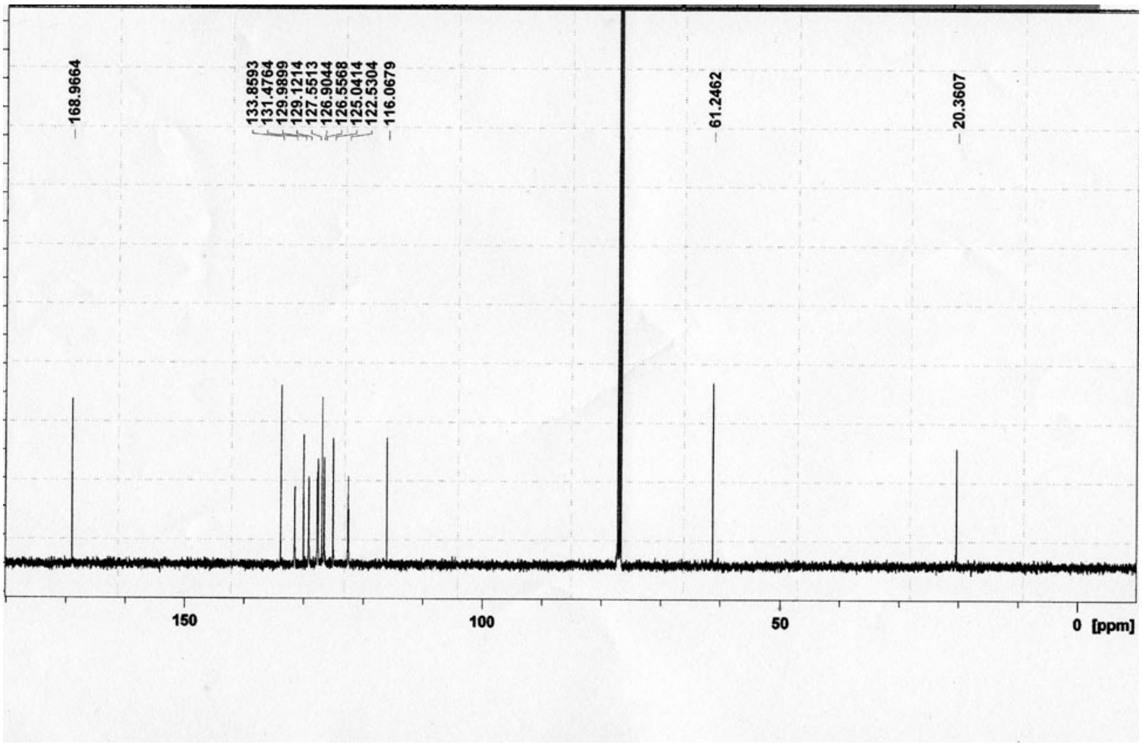

Figure S52. ^{13}C-NMR Spectrum of compound **5f**.

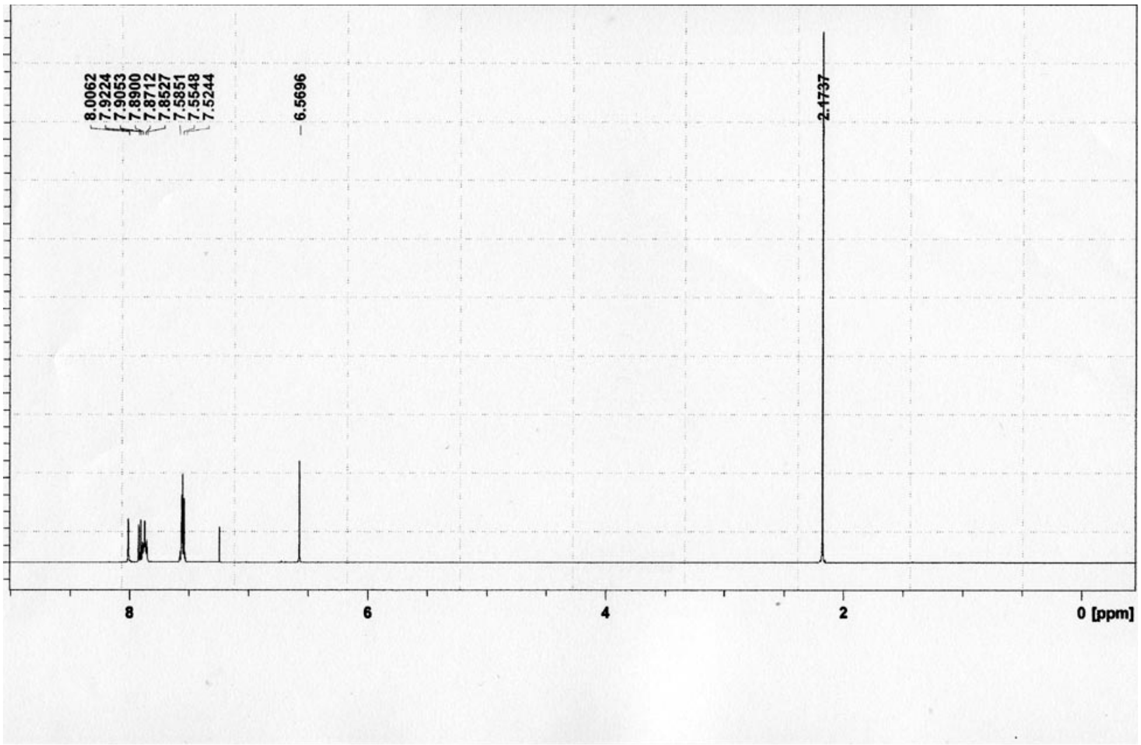

Figure S53. ^{1}H-NMR Spectrum of compound **5g**.

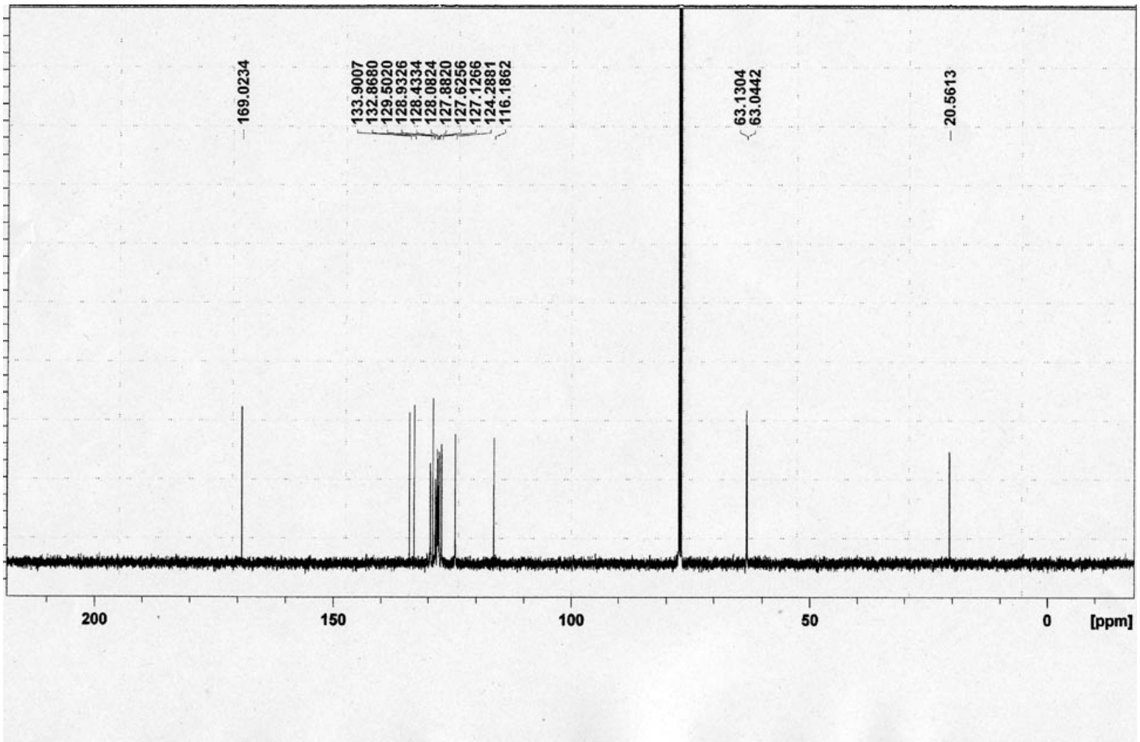

Figure S54. ^{13}C-NMR Spectrum of compound **5g**.

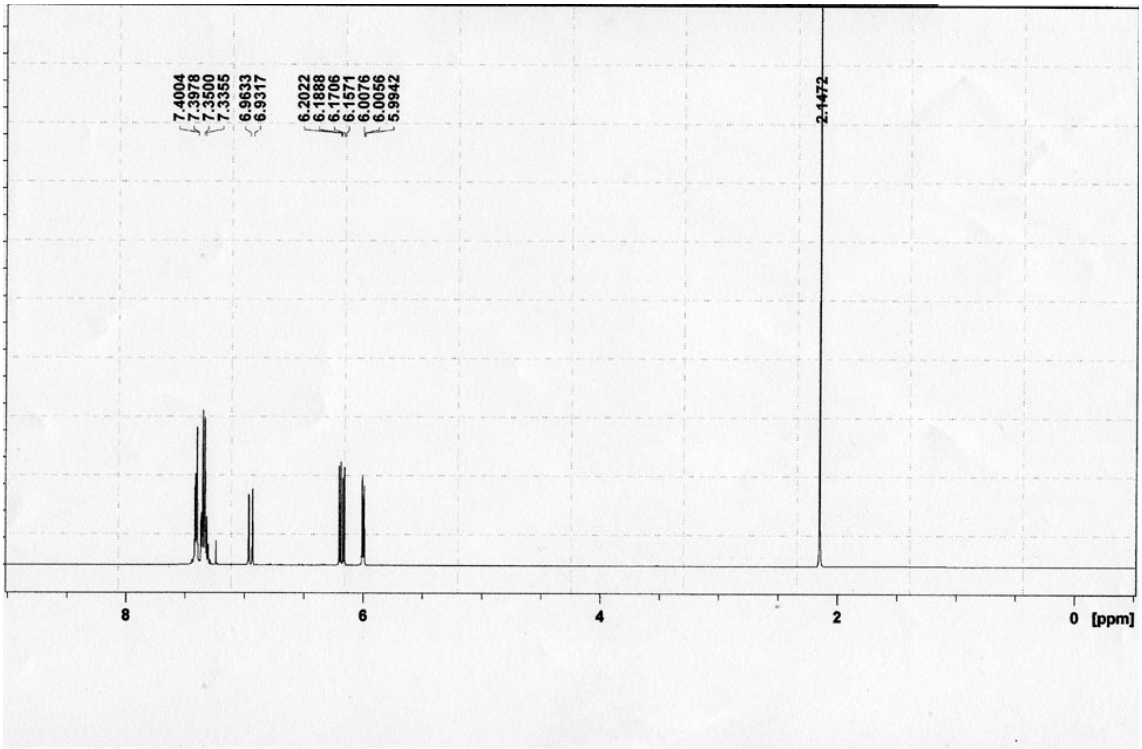

Figure S55. ^{1}H-NMR Spectrum of compound **5h**.

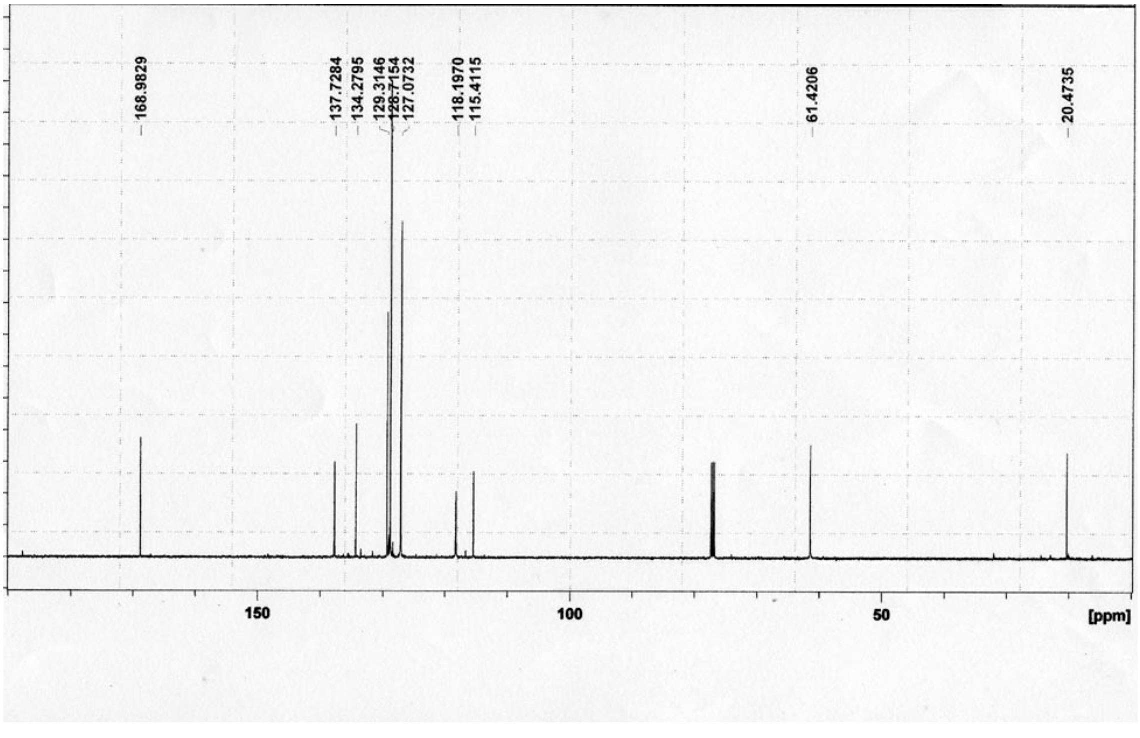

Figure S56. ^{13}C-NMR Spectrum of compound **5h**.

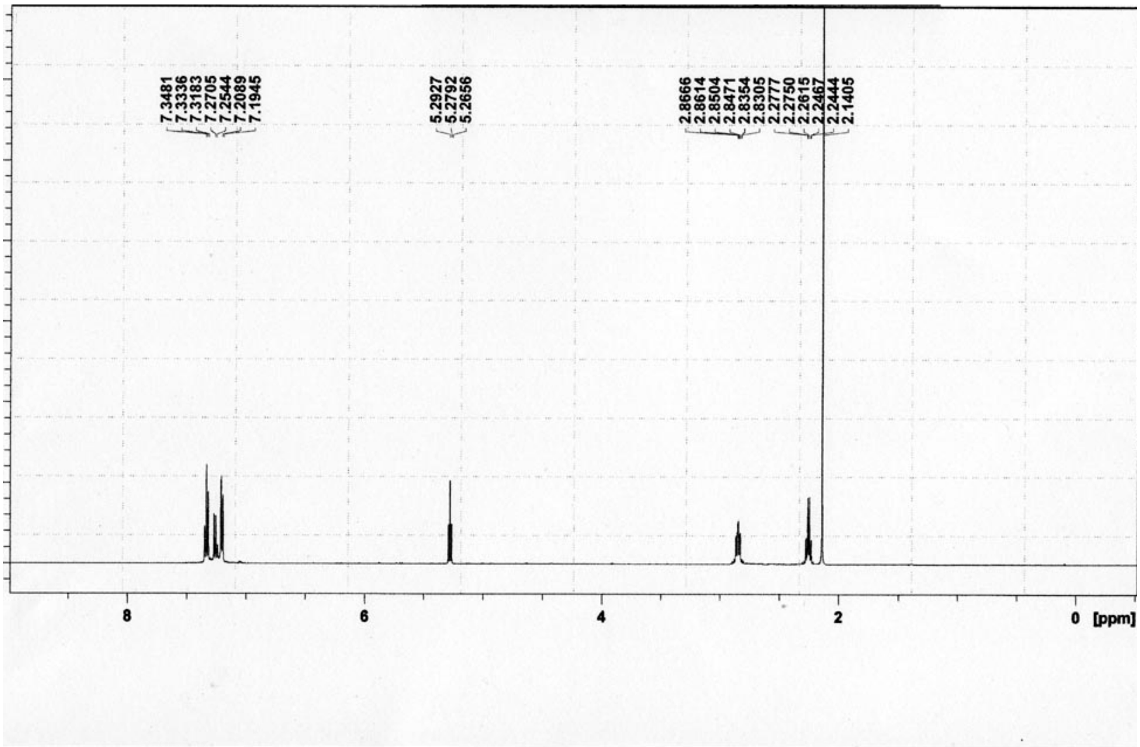

Figure S57. ^{1}H-NMR Spectrum of compound **5i**.

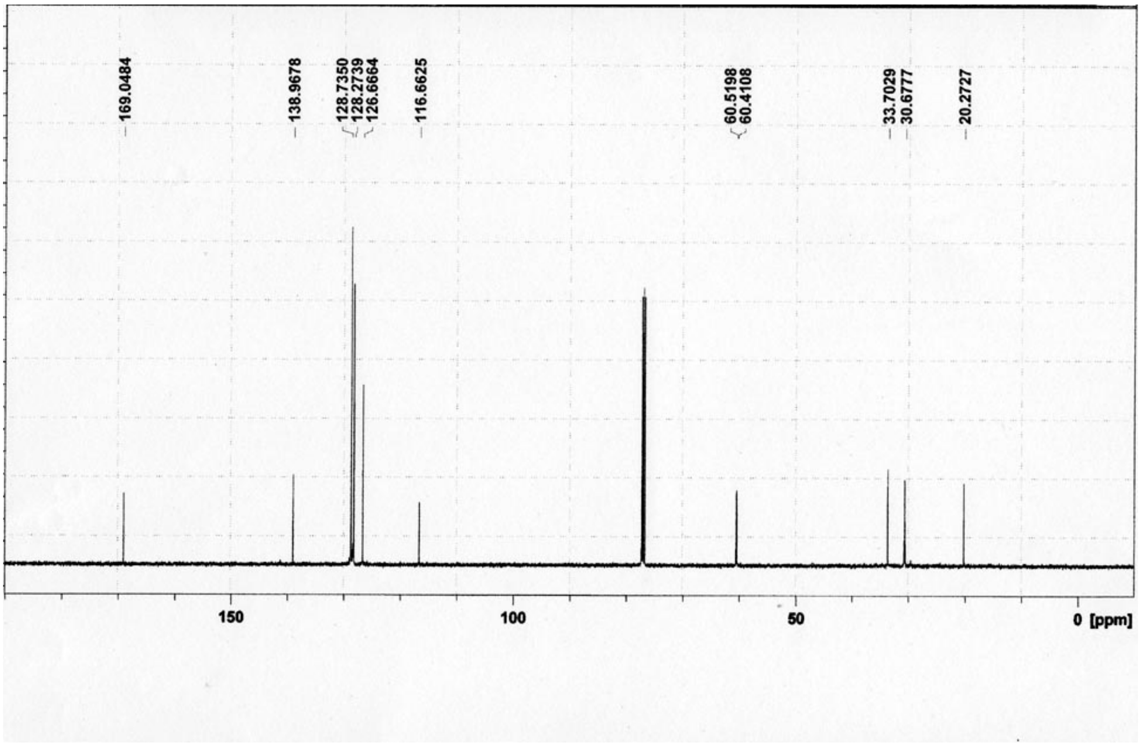

Figure S58. ^{13}C-NMR Spectrum of compound **5i**.

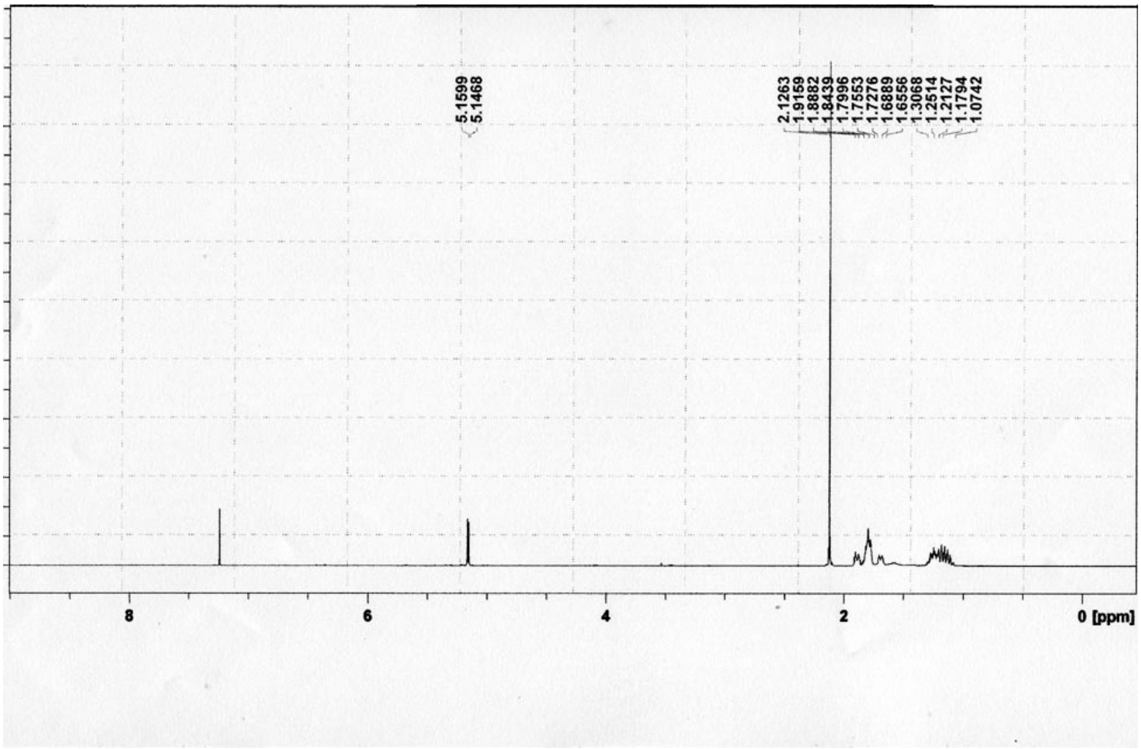

Figure S59. ^{1}H-NMR Spectrum of compound **5j**.

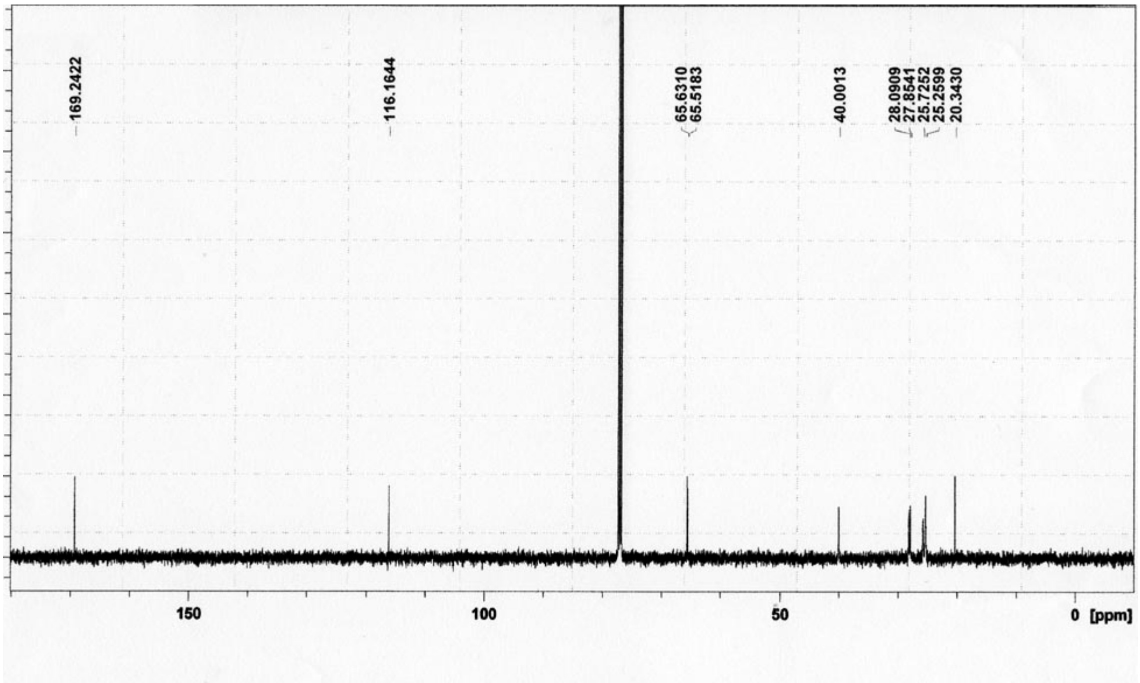

Figure S60 ^{13}C-NMR Spectrum of compound **5j**.

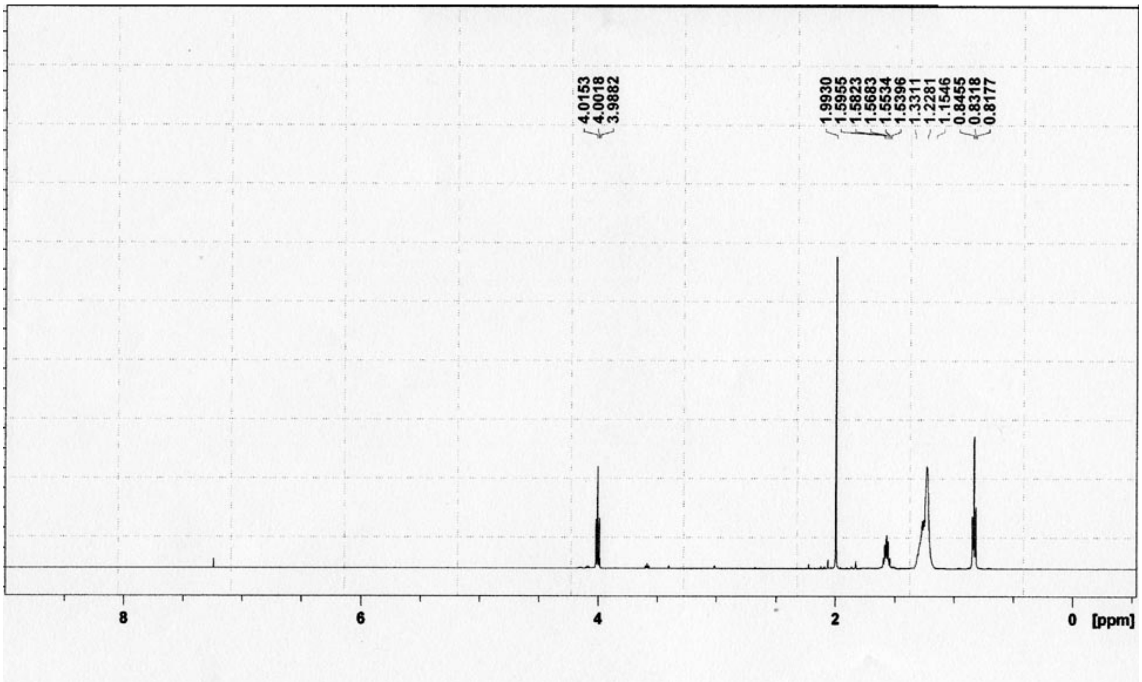

Figure S61. ^{1}H-NMR Spectrum of compound **5k**.

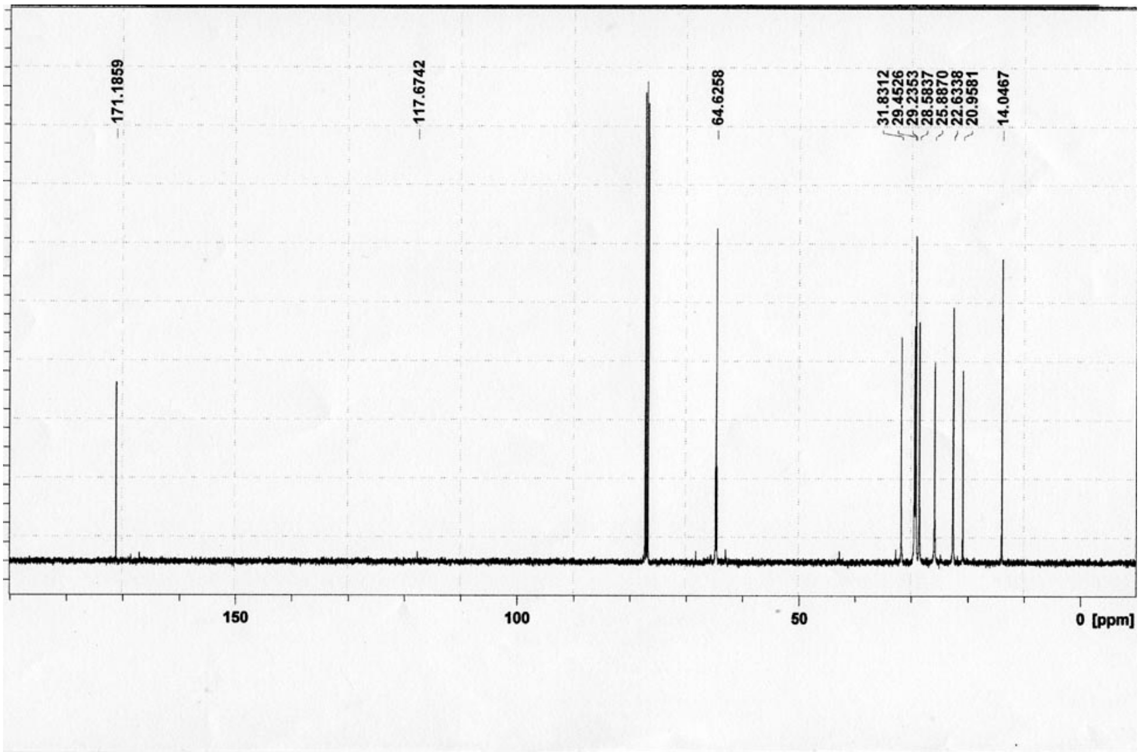

Figure S62. ^{13}C-NMR Spectrum of compound **5k**.

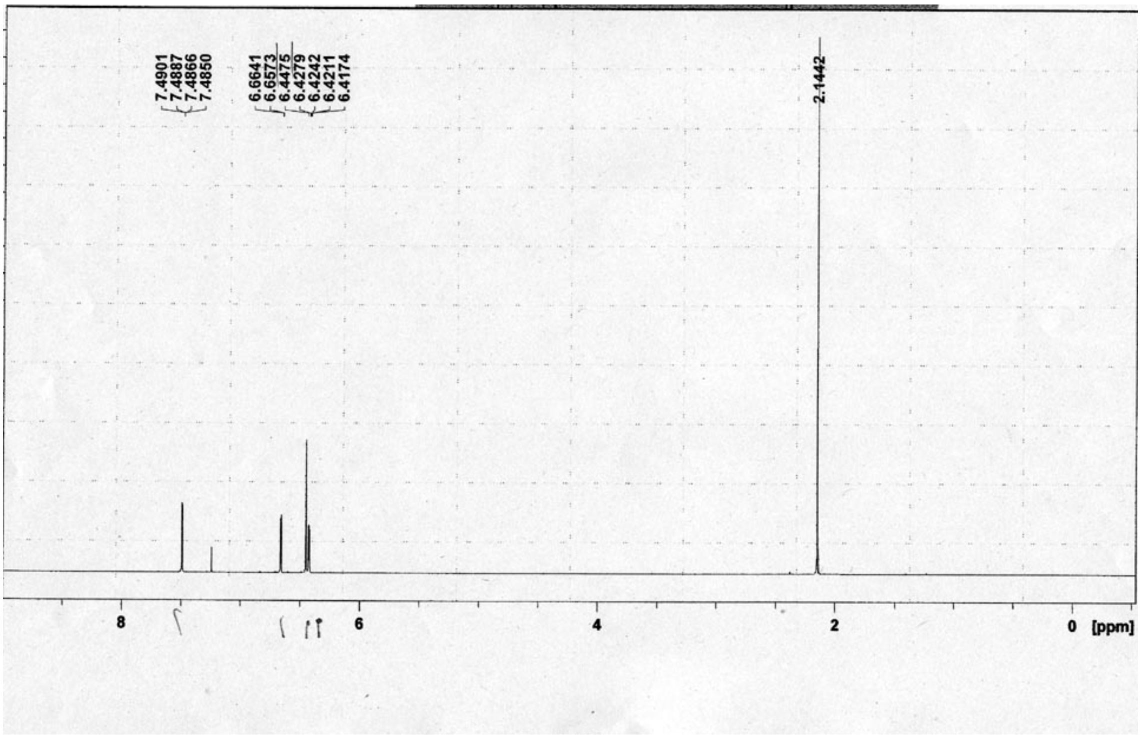

Figure S63. ^{1}H-NMR Spectrum of compound **5l**.

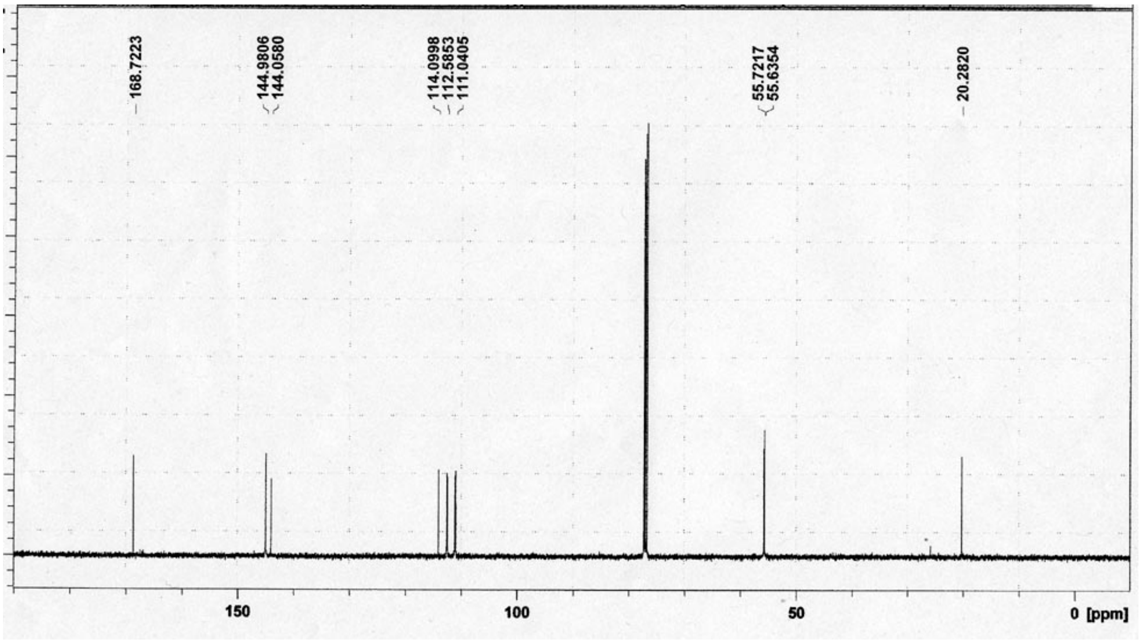

Figure S64. ^{13}C-NMR Spectrum of compound **5l**.
